# Autologous T-Cell-Free Antigen Presentation System Unveils hCMV-Specific NK Cell Response

**DOI:** 10.3390/cells13060530

**Published:** 2024-03-17

**Authors:** Maria O. Ustiuzhanina, Maria A. Streltsova, Nikita D. Timofeev, Maxim A. Kryukov, Dmitriy M. Chudakov, Elena I. Kovalenko

**Affiliations:** 1 Shemyakin and Ovchinnikov Institute of Bioorganic Chemistry, Russian Academy of Sciences, 117997 Moscow, Russia; mashaust1397@gmail.com (M.O.U.); mstreltsova@mail.ru (M.A.S.); ndtimofeev@gmail.com (N.D.T.); chudakovdm@gmail.com (D.M.C.); 2 Ecole Polytechnique Federale de Lausanne, 1015 Lausanne, Switzerland; mkriukov.job@gmail.com; 3 Institute of Translational Medicine, Pirogov Russian National Research Medical University, 117997 Moscow, Russia; 4 Central European Institute of Technology, Masaryk University, 60200 Brno, Czech Republic; 5 Abu Dhabi Stem Cells Center, Abu Dhabi, United Arab Emirates

**Keywords:** memory NK cells, hCMV, NKG2C, HLA-E, cytokines, IFNγ, ERK1/2, RNAseq

## Abstract

NK cells play a decisive role in controlling hCMV infection by combining innate and adaptive-like immune reactions. The hCMV-derived VMAPRTLFL (LFL) peptide is a potent activator of NKG2C^+^ NK cells. Proposed here is an autologous system of LFL stimulation without T lymphocytes and exogenous cytokines that allows us to evaluate NK-cell hCMV-specific responses in more native settings. In this model, we evaluated LFL-induced IFNγ production, focusing on signaling pathways and the degranulation and proliferation of NK cells orchestrated by microenvironment cytokine production and analyzed the transcriptome of expanded NK cells. NK cells of individuals having high anti-hCMV-IgG levels, in contrast to NK cells of hCMV-seronegative and low-positive donors, displayed increased IFNγ production and degranulation and activation levels and enhanced proliferation upon LFL stimulation. Cytokine profiles of these LFL-stimulated cultures demonstrated a proinflammatory shift. LFL-induced NK-cell IFNγ production was dependent on the PI3K and Ras/Raf/Mek signaling pathways, independently of cytokines. In hCMV-seropositive individuals, this model allowed obtaining NK-cell antigen-specific populations proliferating in response to LFL. The transcriptomic profile of these expanded NK cells showed increased adaptive gene expression and metabolic activation. The results complement the existing knowledge about hCMV-specific NK-cell response. This model may be further exploited for the identification and characterization of antigen-specific NK cells.

## 1. Introduction

Natural killer (NK) cells are traditionally considered to be innate immune cells with antigen-independent immune response. This response is characterized by a balance of signals coming from inhibitory and activating NK-cell receptors, including those that recognize “self” molecules of the human leukocyte antigen (HLA-I) [1]. However, recently, increasing attention has been paid to NK cell subsets that exhibit properties typical for adaptive immunity such as antigen specificity and the ability to form long-lived memory cells characterized by rapid and intense responses upon repeated encounter with the pathogen [2]. The most well-known example comprises memory NK cells associated with human cytomegalovirus (hCMV) infection.

The cytomegalovirus is widespread among adults, and by an older age, the proportion of infected people in certain populations can exceed 90% [3]. hCMV is known to persist in the body life-long by having a latent or non-productive infection program and utilizing various immune evasion strategies [4]. For instance, the hCMV UL40 signal peptide is able to be loaded onto HLA-E and stabilize the expression of this molecule on the surfaces of infected cells [5]. This allows the cells to avoid not only elimination by CD8^+^ cytotoxic T cells by downregulating HLA class-I (HLA-I) expression (which is particularly implemented through the expression of proteins blocking the peptide transporter TAP) [6,7,8] and recognition by CD4^+^ T cells by downregulating HLA-II [9] but also elimination by NK cells expressing NKG2A [5]. The HLA-E molecule loaded with a viral peptide serves as a ligand for this inhibitory receptor, which suppresses the antiviral activity of NK cells [5,10,11]. The polymorphism in the UL40 protein provides at least 41 different peptides stabilizing HLA-E [12,13,14,15,16,17,18]. Of those peptides, the VMAPRTLFL (LFL) peptide presented in HLA-E, has been shown to better activate NK cells than other tested peptides through interaction with the activating receptor NKG2C [12,13]. Apart from being found in hCMV, LFL is an HLA-G leader peptide, the presentation of which has been shown to inhibit NK-cell response through NKG2A [19]. The affinity of peptide-loaded HLA-E and NKG2C binding is ~6-fold lower compared to HLA-E/NKG2A binding [19,20,21]. Nevertheless, hCMV promotes the formation of a pool of hCMV-specific NKG2C^+^ NK cells by interacting with HLA-E [22,23].

Accumulated clonal adaptive-like NKG2C^+^ NK cells also express the carbohydrate antigen CD57, which indicates a high degree of their differentiation, and inhibitory killer cell immunoglobulin-like receptors (KIRs), along with the absence of NKG2A expression [24,25], which positions them as missing self-controllers, checking for the presence of classic HLA-I in the context of seemingly normal HLA-E levels. Such NKG2C+ NK cells, as more differentiated NK cells, are part of the CD56^dim^ subset. They also demonstrate increased levels of HLA-DR typical for activated lymphocytes [25,26]. The data about the functional activity of NKG2C^+^ NK cells have varied in different studies. The cytokine-induced IFNγ production of NKG2C^+^ NK cells are lower than in NKG2C^−^ NK cells while the ADCC degranulation has exhibited reverse associations [27]. Further studies revealed an increase in the degranulation potential and IFNγ production in response to the LFL peptide presented in K562-HLA-E and RMA-S-HLA-E [12,21].

The IFNγ production of NK cells is primarily induced by the activation of the JAK/STAT signaling pathways after the stimulation of cytokine receptors [28]. The stimulation of NK cells with IL-12 and IL-18 is widely used to assess IFNγ production [27,29]. Additionally, the measurement of IFNγ production is applied in peptide stimulation experiments [12,21,30]. The signaling pathways mediated by non-cytokine activating receptors depend on the adapter proteins. The NKG2C receptor is associated with DAP12 [31], which transmits activating signals through the PI3K and Ras/Raf/Mek pathways [32]. However, the direct involvement of those pathways in IFNγ production by NKG2C^+^ NK cells has not been clarified.

Different approaches are currently used to induce the activation and proliferation of NK cells in vitro, most of which involve cytokine stimulation, at least in minimal concentrations [33]. The activity of NK cells can be significantly enhanced by cytokines such as IL-2, IL-12, IL-15, IL-18, and IL-21 [33,34]. While IL-12 and IL-18 are actively produced by antigen-presenting cells in the early stages of inflammation [35,36], IL-2 and IL-21 are produced by T cells during the development of adaptive reactions promoting the production of molecules associated with cytotoxic activity (granzyme B, perforin) [37] and IFNγ production [38]. An essential role of IL-12, but not IL-15 or IL-18, in the development of NKG2C^+^ NK cells was shown in a model of hCMV-infected MRC-5 fibroblasts [39]. Commonly, cell lines such as K562-HLA-E, MRC-5, RMA-S/HLA-E [13,39,40], or soluble HLA-E single-chain trimers [41] are used to model antigen presentation. The usage of cell lines and activating cytokines upon the investigation of NK-cell response in vitro could confound the readout. The ability of NK cells to react to particular peptides, presented on autologous cells without additional exogenous stimulation, remains unknown.

In this work, we approximated the approach to identify hCMV-specific NK cells in an autologous cell in vitro system that is closer to the in vivo conditions. Antigen-presenting cells within the T-cell-depleted mononuclear cell (CD3^neg^PBMC) population used in this system can effectively present exogenous peptides in the HLA-E context. We directly showed that the mere addition of the VMAPRTLFL peptide to CD3^neg^PBMC from hCMV highly seropositive individuals in the absence of co-stimulation with exogenous cytokines induced IFNγ production and the degranulation and proliferation of NK cells in contrast to the group of donors with an absence or low titer of hCMV-specific IgG. These NK cell activation events were accompanied by changing the profile of cytokines endogenously produced in the culture, including IL-12 and IL-10. Furthermore, we analyzed the signaling pathways involved in LFL-induced IFNγ production in this system and performed a transcriptome analysis of highly proliferated NK cells.

## 2. Materials and Methods

### 2.1. Samples

For the experimental work, peripheral blood samples were obtained from healthy volunteers: 34 donors aged from 22 to 65 years. hCMV-specific IgG titers were determined via enzyme-linked immunosorbent assay using a commercial kit (Vector-Best, Novosibirsk, Russia).

### 2.2. Obtaining of Immune Cells

Peripheral blood mononuclear cells (PBMCs) were obtained via gradient centrifugation with Ficoll. PBMCs underwent the positive magnetic separation of CD3^+^ cells to obtain CD3^neg^PBMC (RWD Life Science Co., Shenzhen, China). The resulting CD3^neg^PBMCs were cultured in a medium based on NK-MACS Medium (Miltenyi Biotec, Bergisch Gladbach, Germany) and DMEM (PanEco, Moscow, Russia) in a 1:1 ratio, supplemented with 2 mM L-glutamine, 1 mM sodium pyruvate, 10% fetal bovine serum (FBS, HyClone, Logan, UT, USA), and 1% antibiotic (Antibiotic-Antimycotic Solution, Corning, NY, USA). Cells were cultured in a 96-well round-bottom plate in concentration of 2.5 million cells per mL in a CO_2_ incubator at 37 °C.

### 2.3. Peptide Synthesis

Solid-phase syntheses of peptides VMAPRTLFL and VMAPQSLLL (LFL and LLL, respectively) [12] were carried out using a custom-made automated parallel peptide synthesizer based on the Gilsonon Gilson liquid handler. Fmoc strategy with HATU/DIPEA (GL Biochem Shanghai, China) activation was applied. The C-terminal amino acid was attached to the 2-chlorotrityl chloride-activated resin in the presence of DIPEA for 2 h. After that, all residues were coupled automatically via the following cycled procedure: PIP (30% solution in NMP, 25 mL for 1 g of resin), 2 × 10 min; DMF (20 mL for 1 g of resin), 5 × 5 min; Fmoc-AA(PG)-OH (8 eq., 0.5 M solution in NMP)/HATU (8 eq., 0.5 M solution in NMP)/DIPEA (16 eq.), 40 min; and DMF (25 mL for 1 g of resin), 5 × 5 min. After the synthesis was completed, the resin was washed with MeOH (30 mL for 1 g of resin, 5 × 5 min) and dried under reduced pressure. The following reagents were used—TIS (Iris Biotech, Marktredwitz, Germany), 4-methylpiperidine (Mosinter Chemicals, Zhejiang, China), trifluoroacetic acid (Solvay S.A., Brussels, Belgium), and acetonitrile (gradient-grade, Biosolve, Dieuze, France). DCM was dried over CaH2 and refluxed before use. All other reagents and solvents were purchased from local manufacturers and used without further purification.

For final deprotection of the peptide, a standard cleavage mixture was used (TFA/DTT/H2O/TIS 89/5/5/1). Dry peptidyl resin was suspended in the cleavage mixture (15 mL for 1 g of resin) and was shaken for 2 h. After the filtration of the resin, diethyl ether (tenfold excess corresponding to the cleavage mixture volume) was added and suspension was left to stand at −20 °C for a couple of hours, then centrifuged, washed 3 times with diethyl, and dried under reduced pressure. The structure of the peptide was confirmed through mass spectrometric analysis using the Dionex UltiMate 3000 HPLC chromatographic system (Thermo Scientific, Waltham, MA, USA) and an LTQ-Orbitrap Velos mass spectrometer (Thermo Scientific, Waltham, MA, USA).

Next, the peptides were diluted in water and used for experiments at a final concentration of 300 μM. The peptides were tested to induce the surface expression of HLA-E. CD3^neg^PBMCs were incubated with the LFL/LLL peptide or without peptides for 6 h. After the incubation, surface staining was conducted using fluorescently labeled monoclonal antibodies CD3-FITC (Beckman Coulter, Brea, CA, USA), HLA-E-PE, CD14-PE-Vio770, and CD56-APC (Miltenyi Biotec, Bergisch Gladbach, Germany).

### 2.4. Functional Tests

Cytometric measurement of intracellular IFNγ levels in NK cells was performed in two replicates for each donor. CD3^neg^PBMCs were incubated with the LFL/LLL peptide or without peptides for 6 h with brefeldin A. The positive control was obtained after the incubation with IL-12 (10 ng/mL) and IL-18 (20 ng/mL). After the incubation, surface staining was conducted using fluorescently labeled monoclonal antibodies CD56-Brilliant Violet 421 (Sony, San Jose, CA, USA), CD3-PE-Vio770, CD57-APC-Vio770, NKG2C-APC, and KIR2DL2/DL3-PE (Miltenyi Biotec, Bergisch Gladbach, Germany). A cell fixation/permeabilization kit (internal staining kit, Miltenyi Biotec, Bergisch Gladbach, Germany) was then used, followed by staining with anti-IFNγ antibodies (IFNγ-FITC, Miltenyi Biotec, Bergisch Gladbach, Germany).

The level of NK cell degranulation was analyzed in two replicates for each donor after a 16 h incubation with the LFL peptide by assessing the lysosomal marker LAMP-1 (CD107a). Brefeldin A and CD107a-PE-Cy5 antibody (Sony, San Jose, CA, USA) were added 6 h before the measurement. After the incubation, the surface staining was conducted with previously specified antibodies and HLA-DR-FITC antibody (Miltenyi Biotec, Bergisch Gladbach, Germany).

Samples were analyzed using the MACSQuant 10 flow cytometer (Miltenyi Biotec, Bergisch Gladbach, Germany) equipped with lasers λ = 405, λ = 488, and λ = 635 nm.

### 2.5. Inhibition of NK Cells with Transcription Factor Inhibitors

The following transcription factor inhibitors were used: fludarabine phosphate (Sigma-Aldrich, St. Louis, MO, USA)—STAT1 inhibitor, FR 180204 (Sigma-Aldrich, St. Louis, MO, USA)—ERK1/2 inhibitor, LY-294, 002 hydrochloride (Sigma-Aldrich, St. Louis, MO, USA), and cryptotanshinone (Tocris Bioscience, Bristol, UK)—STAT3 inhibitor. All substances were diluted in DMSO (AppliChem, Darmstadt, Germany) to the following concentrations: fludarabine phosphate—0.1, 1, and 10 μg/mL; FR 180204—0.5, 5, and 50 μg/mL; LY-294, 002 hydrochloride—0.1, 1, and 10 μg/mL, cryptotanshinone—0.1, 1, and 10 μg/mL. The titration of inhibitors was performed. Next, inhibitors were added to freshly isolated CD3^neg^PBMC in the presence of the LFL peptide. After 6 h, NK cells were labeled with fluorescent antibodies for the following intracellular IFNγ assay.

### 2.6. Cytokine Measurements

CD3^neg^PBMCs were incubated in a complete medium with the LFL peptide for 12 h. The supernatants were extracted and assessed via flow cytometry for the production of 12 soluble cytokines in one sample (GM-CSF, IFN-α, IFN-γ, IL-2, IL-4, IL-5, IL-6, IL-9, IL-10, IL-12p70, IL-17A, and TNF-α) using the MACSPlex Cytokine 12 fluorescent bead kit (Miltenyi Biotec, Bergisch Gladbach, Germany) according to the manufacturer’s instructions.

### 2.7. Proliferation Test

Freshly isolated CD3^neg^PBMCs were stained with 5 μM CFSE (Molecular Probes, Eugene, OR, USA) in 100 μL of PBS (PanEco, Moscow, Russia) and subsequently incubated for 15 min at 37 °C and protected from light. CD3^neg^PBMCs were washed three times in RPMI-1640 (PanEco, Moscow, Russia) supplemented with 10% FBS (Hyclone, Logan, UT, USA). CFSE^high^ CD3^neg^PBMCs were cultivated in two replicates for each donor for 7 days in medium. The changing of medium was conducted on the 4th day of cultivation. The control samples were cultivated without any stimulation while other samples had LFL added at 0th and 4th days. To exclude the analysis of dead cells, the dye Sytox-VioBlue (Invitrogen, Burlington, ON, USA) was used.

### 2.8. CFSE^low^ NK Cells Cultivation and Collection for RNA Sequencing

Highly proliferating NK cells (CFSE^low^CD56^+^CD3^−^Sytox^−^), after 7 days of stimulation with the LFL peptide, were isolated in some experiments using cell sorting on a FACSVantage DiVa (Beckton Dickinson, Franklin Lakes, NJ, USA) and placed in a 96-well plate of 100 cells each for further cultivation. Those cells were stimulated with IL-2 (Hoffmann La-Roche, Basel, Switzerland) at 100 units/mL and K562 feeder cells expressing membrane-bound IL-21 (K562-mbIL21) [42]. The expansion rates of those cells were assessed 10 days after sorting. Before restimulation, NK cells were incubated in a medium without IL-2 for 24 h. Then CD3^neg^PBMCs of the same donor as NK cells were thawed and stained with CFSE to distinguish these cells in the following cytometric assays. CFSE^high^CD3^neg^PBMCs were incubated with LFL/LLL without any peptide for 2 h; then, they were added to NK cells (1:2 ratio) for 6 and 16 h to perform IFNγ and degranulation assays, respectively.

Highly proliferating NK cells (CFSE^low^CD56^+^CD3^−^Sytox^−^) of donor 32, in 3 replicates, were collected in the RLT lysis buffer (QIAGEN, Hilden, Germany) for the RNA sequencing. 

### 2.9. RNA Preparation and Sequencing

Total RNA was extracted from collected samples with TRIzol (Invitrogen, Carlsbad, CA, USA). Treatment with DNase was performed to avoid contamination by genomic DNA (Thermo Fisher Scientific, Waltham, MA, USA). The following conversion of total RNA into cDNA was performed using NebNext Single Cell/Low Input RNA Library Prep Kit for Illumina (New England Biolabs, Ipswich, MA, USA) according to the manufacturer’s instructions. Adaptor ligation and addition of indexes were conducted using NEBNext Multiplex Oligos for Illumina (New England Biolabs, Ipswich, MA, USA). The final average fragment size was 300 bases. Each library was then subjected to paired-end sequencing on Illumina NovaSeq6000 by the Genomic Core Facility of Skolkovo Institute of Science and Technology.

### 2.10. Sequence Analysis

Paired-end RNA-sequencing reads were aligned to the human reference genome (hgGRCh38/ensembl.org) with subsequent unique-gene hit-count calculation using Kallisto [43]. DESeq2 was used to analyze the data and perform a differential expression assay [44]. Genes with an absolute log_2_- fold change (LFC) > 0.58 and *P*adj < 0.2 were considered as differentially expressed genes (DEGs) for each comparison. Enrichment analysis was performed using Gene ontology (GO) analysis from *clusterProfiler* package. GSEA was run using the gseGO function with *p*-adjusted values obtained via Benjamini–Hochberg method [45].

### 2.11. Statistical Analysis

Data were analyzed using Microsoft Excel, FlowJo (FlowJo X, OR, Ashland, USA), and GraphPad Prism (version 8.4.3, GraphPadSoftware, Boston, MA, USA). Figures represent means ± standard deviations; statistical analysis was performed using Wilcoxon test for paired nonparametric samples unless otherwise stated. A *p* value < 0.05 was considered statistically significant.

## 3. Results

### 3.1. Two Groups of Donors May Be Identified Based on the Cluster Analysis of IgG Serum Level and Surface Marker Expression

**Figure 1:**
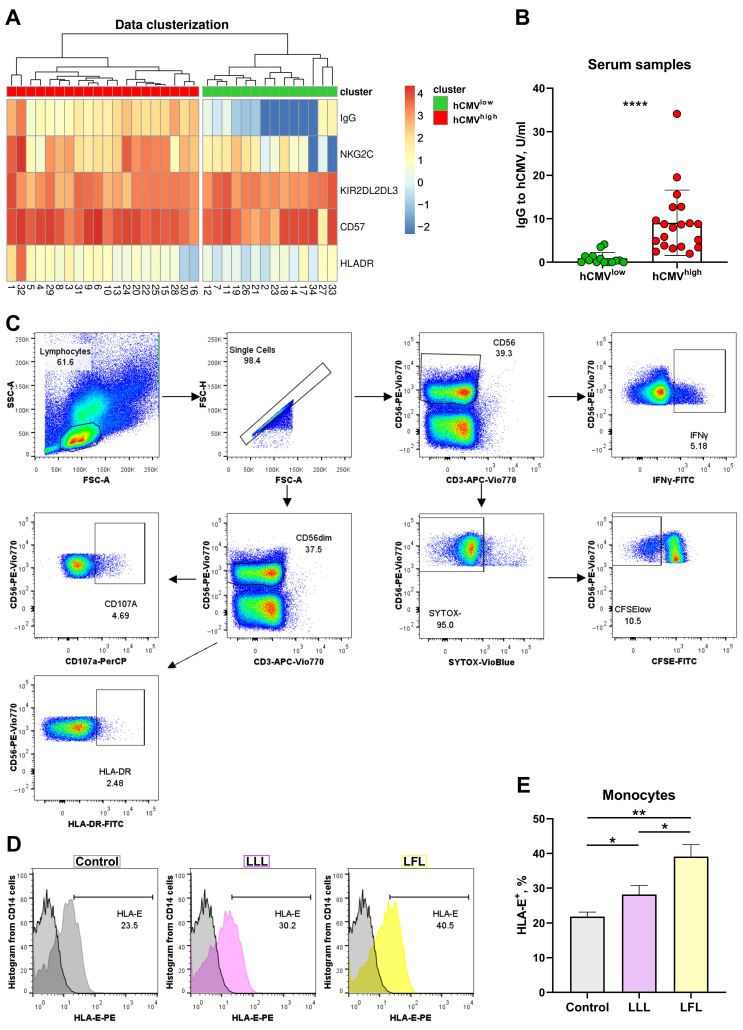
Cluster analysis of NK cell phenotype based on hCMV-specific IgG serum levels. (**A**) Hierarchical clustering of log-normalized anti-HCMV IgG titers and percentages of HLA-DR^+^, NKG2C^+^, CD57^+^, and KIR2DL2/DL3^+^ NK cells for 34 individuals using a heatmap. Two discrete sample-level clusters are depicted. (**B**) Anti-hCMV IgG antibody titers in units/mL. (**C**) Gating scheme of cytometric analysis of NK cells in CD3^neg^PBMC samples, presented as pseudocolures (colors corresponds to the density). (**D**) Representative cytometric data on HLA-E surface expression without and after stimulation with LLL/LFL on monocytes (CD14^+^). (**E**) Comparison of HLA-E surface expression on monocytes in control samples and samples stimulated with LLL or LFL (*n* = 4). The color of the boxes corresponds to the stimulation conditions, where gray—no stimulation, light yellow—stimulation with the LFL peptide (VMAPRTLFL), and light purple—stimulation with LLL peptide (VMAPQSLLL). Data are presented as means ± SDs. The paired Wilcoxon test was used to analyze the data (**B**), and the One-way ANOVA was used for multiple comparisons (**E**). * *p* < 0.05, ** *p* < 0.01, and **** *p* < 0.0001.

Surface expression levels of NKG2C, HLA-DR, CD57, and KIR2DL2/DL3 in NK cells were analyzed in PBMC samples obtained from 34 healthy adult volunteers via flow cytometry. NK cells were gated as CD56^+^CD3^−^ cells. The hCMV serological status was determined. Pre-log normalized data on anti-hCMV IgG antibody titers together with the proportions of the NKG2C-, HLA-DR-, CD57-, and KIR2DL2/DL3-expressing NK cells were used for clustered heatmap analysis with hierarchical relationships between samples in order to divide the donors into groups. Two major clusters were identified in the analyzed donor cohort (Figure 1A) based on the serum levels of anti-hCMV IgG antibodies and proportions of surface markers according to hierarchical clustering. The first cluster, characterized by high and medium titers of IgG-hCMV antibodies, was designated as hCMV^high^. The second cluster corresponded to the hCMV-seronegative status, together with the low hCMV seropositivity of donors (Figure 1A,B).

The ability of CD3^neg^PBMC to present peptides was analyzed in a preliminary study. Spontaneous HLA-E expression was observed on the surfaces of monocytes in CD3^neg^PBMCs whereas NK cells almost did not express HLA-E at detectable levels (Figure 1D,E, Supplementary Figure S1). Both peptides, LFL and LLL, increased HLA-E levels in monocytes and induced the HLA-E surface expression in NK cells, although to a lower level compared to monocytes. The addition of LFL led to higher HLA-E levels in both monocytes and NK cells compared to LLL (Figure 1D,E, Supplementary Figure S1).

### 3.2. IFNγ Production Induced in NK Cells from hCMV^high^ Group by the LFL Peptide Depends on the NKG2C Level

**Figure 2:**
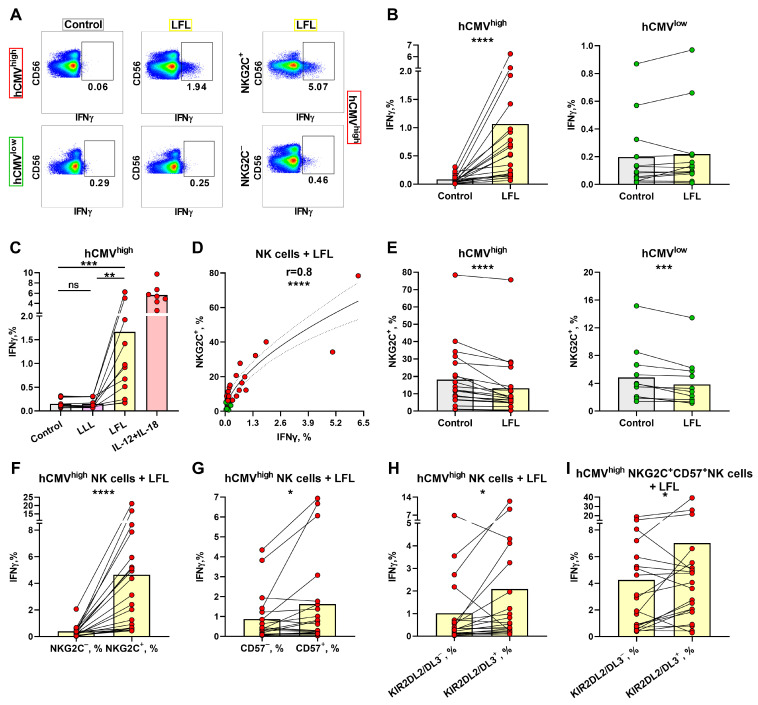
IFNγ production by NK cells upon stimulation with the LFL peptide. (**A**) Representative cytometric data on IFNγ production levels in samples of one hCMV^high^ and one hCMV^low^ donor without and after stimulation with LFL in total population of NK cells and in NKG2C^+^ NK cells. (**B**) Comparison of NK-cell IFNγ production in control (unstimulated) samples and samples stimulated with LFL for hCMV^high^ (*n* = 20) and hCMV^low^ (*n* = 14) donor groups. (**C**) Comparison of NK-cell IFNγ production in control samples and samples stimulated with LLL or LFL in a group of hCMV^high^ donors (*n* = 11) and positive controls stimulated with IL-12 and IL-18 (*n* = 6). (**D**) Spearman correlation analysis of NKG2C expression and IFNγ production in NK cells stimulated with LFL in all studied donors, presented using a nonlinear regression model (*n* = 34). (**E**) The proportion of NKG2C^+^ NK cells in control samples and samples stimulated with LFL in hCMV^high^ (*n* = 20) and hCMV^low^ (*n* = 13) donors. (**F**) Comparison of IFNγ production in NKG2C^+^ and NKG2C^−^ NK cells in samples stimulated with LFL in the hCMV^high^ group (*n* = 20). (**G**) Comparison of IFNγ production by CD57^+^ and CD57^−^ NK cells in samples stimulated with LFL in the hCMV^high^ group (*n* = 20). (**H**) Comparison of IFNγ production by KIR2DL2/DL3^+^ and KIR2DL2/DL3^−^ NK cells in samples stimulated with LFL in the hCMV^high^ group (*n* = 20). (**I**) Comparison of IFNγ production by CD57^+^NKG2C^+^KIR2DL2/DL3^−^ and CD57^+^NKG2C^+^KIR2DL2/DL3^+^ NK cells in samples stimulated with LFL in the hCMV^high^ group (*n* = 20). The colors of the symbols correspond to the groups, wherein green is hCMV^low^ and red is hCMV^high^. The colors of the boxes correspond to the stimulation conditions, wherein gray—no stimulation, light yellow—stimulation with the LFL peptide (VMAPRTLFL), light purple—stimulation with LLL peptide (VMAPQSLLL), and pink—positive control IL-2 + IL-18. Data are presented as mean values with lines connecting symbols for each donor. Each symbol represents the mean of two replicates. The paired Wilcoxon test was used to analyze the data, and the Friedman paired test was used for multiple comparisons. Ns (not significant) *p* > 0.05, * *p* < 0.05, ** *p* < 0.01, *** *p* < 0.001, and **** *p* < 0.0001.

To identify the differences in the responses of NK cells to LFL depending on the hCMV serological statuses of individuals, the levels of IFNγ-producing NK cells in two donor groups were measured. Since NKG2C^+^ T cells are also able to interact with the LFL peptide [46], we evaluated intracellular IFNγ production by NK cells in CD3^neg^PBMCs upon stimulation with the LFL peptide for 6 h without any exogenous addition of cytokines. Even though CD56^bright^ NK cells are involved in cytokine production, we observed a dominant IFNγ production in the CD56^dim^ NK cell subpopulation (Figure 2A, Supplementary Figure S2A). Due to the minimal or absent involvement of CD56^bright^ NK cells in IFNγ production in response to LFL, we determined the IFNγ production in the total population of NK cells. The gating scheme is presented in Figure 1C. NK cells from the hCMV^high^ group showed a statistically significant increase in IFNγ production upon the addition of the LFL peptide (Figure 2A,B). In contrast, NK cells from the hCMV^low^ group did not show statistically significant changes in IFNγ production after the addition of LFL compared to control samples that were not stimulated (Figure 2A,B). Additional experiments were conducted in order to analyze the IFNγ production by NK cells of hCMV^high^ individuals, where the inactive peptide VMAPQSLLL (LLL) was used as a biological control. The addition of LLL did not result in an increase in IFNγ production by NK cells (Figure 2C) compared to unstimulated controls. Thus, the LFL peptide in the absence of exogenous cytokine stimulation and endogenous stimulation by CD3^+^ cells induced the IFNγ production by NK cells in the hCMV^high^ donor group. 

A positive correlation was found between the proportions of NKG2C^+^ NK cells and NK-cell IFNγ production levels in samples stimulated with the LFL peptide (Figure 2D). A slightly weaker but significant positive correlation was revealed between the proportions of CD57^+^ and IFNγ^+^ NK cells, but only in the hCMV^high^ group (Supplementary Figure S2B). No association between IFNγ production and the proportion of KIR2DL2/DL3^+^ NK cells was identified (Supplementary Figure S2C). In the analysis of the NK cell surface markers, a decrease in the proportion of NKG2C^+^ NK cells was observed after cultivation in the presence of LFL (Figure 2E). Interestingly, the group of hCMV^low^ individuals revealed the same decrease in the proportion of NKG2C^+^ NK cells (Figure 2E).

Next, we performed an analysis of the ability of different NK cell subsets to produce IFNγ in response to LFL. NKG2C^+^ NK cells produced significantly more IFNγ compared to NKG2C^−^ NK cells in the hCMV^high^ group (Figure 2F). The hCMV^low^ group also showed slightly higher IFNγ production by NKG2C^+^ NK cells in response to LFL; however, half of the donors had similar IFNγ production when comparing NKG2C^+^ and NKG2C^−^ NK cells. Supposedly, the minor increase in IFNγ production was mediated by the NK cell reactions of donors with low hCMV seropositivity (Supplementary Figure S2D). Higher IFNγ production levels were also observed in the CD57^+^ and KIR2DL2/DL3^+^ NK cell subsets compared to their negative counterparts in the hCMV^high^ group (Figure 2G,H). However, in some individuals from this group, NK cells oppositely displayed a decreased IFNγ-producing cell proportion among the KIR2DL2/DL3^+^ subset compared to the KIR2DL2/DL3-negative cells. In accordance with the higher and lower IFNγ production levels in the KIR2DL2/DL3^+^ subset, the samples were divided into additional subgroups 1 and 2, correspondingly. Subgroup 1 showed significantly lower proportions of IFNγ-producing cells in more differentiated CD57^+^ NK cells, and subgroup 2, in contrast, was characterized by higher IFNγ production levels in these mature CD57^+^ NK cells, in comparison with the KIR2DL2/DL3-positive cell subset (Supplementary Figure S2E). At the same time, no differences were found in the proportions of NKG2C-, KIR2DL2/DL3-, and CD57-expressing NK cells between those subgroups (Supplementary Figure S2F).

Since NKG2C^+^-adaptive NK cells are typically highly differentiated cells (CD57^+^ cells) [25,47], the proportion of IFNγ-producing cells was compared, in this NKG2C^+^CD57^+^ subset, between KIR2DL2/DL3^+^ and KIR2DL2/DL3^−^ NK cells. We observed an increased IFNγ production level in CD57^+^NKG2C^+^KIR2DL2/DL3^+^ NK cells (Figure 2I). Thus, in individuals with mostly more pronounced B-cell hCMV-specific responses (hCMV^high^), the IFNγ production response to the LFL peptide was largely confined to NKG2C^+^ NK cells, which partly represented the highly differentiated CD57^+^KIR-expressing NK cell subset.

### 3.3. ERK1/2 and PI3K Are Directly Involved in the IFNγ Production by NK Cells in Response to the LFL Peptide in the CD3^neg^PBMC In Vitro System

**Figure 3:**
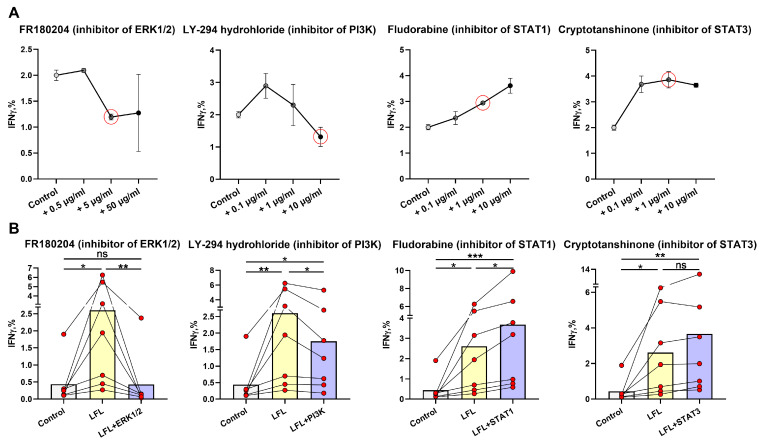
Analysis of the participation of intracellular signaling molecules in NK-cell IFNγ production induced by the LFL peptide. (**A**) Titration of the ERK1/2 (FR180204), PI3K (LY-294,002 hydrochloride), STAT1 (Fludorabine), and STAT3 (Cryptotanshinone) inhibitors for determining the working concentrations (*n* = 3). The red circles represent the chosen inhibitors concentrations. (**B**) Effects of ERK1/2 (FR180204), PI3K (LY-294,002 hydrochloride), STAT1 (Fludorabine), and STAT3 (Cryptotanshinone) inhibitors at the concentrations indicated in (**A**) on the level of IFNγ production in NK cells of hCMV^high^ donors (*n* = 7). Data are presented as mean values with lines connecting symbols for each donor. Each symbol represents the mean of two replicates. The paired Friedman statistical test was used to analyze the data. Ns *p* > 0.05, * *p* < 0.05, ** *p* < 0.01, *** *p* < 0.001.

To assess signaling pathways involved in NK-cell IFNγ production in response to the LFL peptide in the used autologous model, we performed a series of NK cell activation experiments in the presence of selective inhibitors of STAT1 (Fludorabine), STAT3 (Cryptotanshinone), ERK1/2 (FR180204), and PI3K (LY-294,002 hydrochloride). The concentrations of the inhibitors were tested based on literature recommendations [48,49] and preliminary experiments. The following inhibitor concentrations were selected as working concentrations for the following experiments: 1 μg/mL for fludarabine phosphate and cryptotanshinone, 10 μg/mL for LY-294,002 hydrochloride, and 5 μg/mL for FR180204 (Figure 3A).

To study the signaling pathways involved in NK-cell IFNγ production induced by LFL, we have chosen seven donors from the hCMV^high^ group, which showed the most significant increase in IFNγ levels after the incubation with LFL (Figure 2B).

The addition of the ERK1/2 inhibitor resulted in a significant decrease in IFNγ production induced by LFL, and there was no difference between unstimulated samples and LFL-stimulated samples treated with the inhibitor (Figure 3B). Thus, the activation of the Ras/Raf/Mek/ERK1/2 signaling pathway is required for the LFL-induced IFNγ production by NK cells in this system.

The PI3K inhibitor led to a decrease in the NK-cell IFNγ production in response to the LFL peptide; however, still, the level of IFNγ in the presence of the inhibitor was higher than in control samples (Figure 3B). This indicates that in these settings, some other compensatory mechanisms may still activate IFNγ production, but at a lower level than with functional PI3K.

The addition of fludarabine, the inhibitor of the transcription factor STAT1, significantly increased the IFNγ production by NK cells compared to both unstimulated samples and those stimulated with the LFL peptide (Figure 3B). Presumably, the inhibition of the JAK1-STAT1 signaling pathway may affect the IFNγ production by NK cells by involving compensatory pathways, for example, Ras/Raf/Mek/ERK1/2.

The STAT3 inhibitor cryptotanshinone did not cause a significant inhibition of IFNγ production in NK cells stimulated with LFL (Figure 3B). Furthermore, the simultaneous addition of both inhibitors of STAT1 and STAT3 had no effects on IFNγ production by NK cells in response to LFL (Supplementary Figure S3). The absence of an inhibitory effect indicates that the JAK1/3-STAT1/3 signaling pathway is not involved in the IFNγ response of NK cells to the LFL peptide in these settings.

### 3.4. NK Cell Degranulation and Activation Is Increased in Response to the LFL Peptide

**Figure 4:**
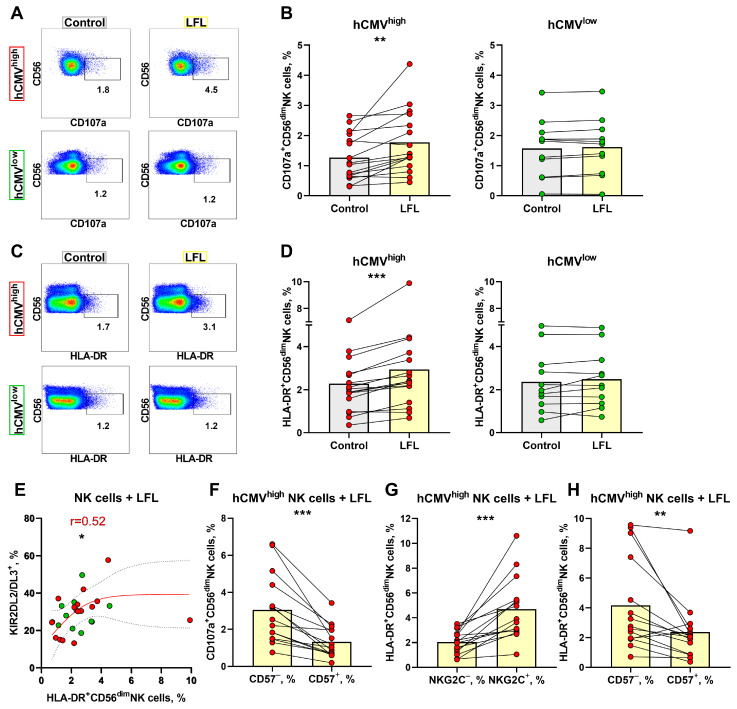
Degranulation and activation of NK cells in response to LFL. (**A**) Representative cytometric data on the CD107a surface levels in NK cells in samples of one hCMV^high^ and one hCMV^low^ donor without and after stimulation with LFL. (**B**) Comparison of the CD56^dim^ NK cell degranulation levels in control samples and in samples stimulated with LFL in hCMV^high^ (*n* = 16) and hCMV^low^ donors (*n* = 11). (**C**) Representative cytometric data on HLA-DR expression level in samples of one hCMV^high^ and one hCMV^low^ donor without stimulation and after stimulation with LFL. (**D**) Comparison of CD56^dim^ NK cell activation levels in control and samples stimulated with LFL in hCMV^high^ (*n* = 16) and hCMV^low^ donors (*n* = 11). (**E**) Spearman correlation analysis of KIR2DL2/DL3 expression and level of HLA-DR expression by CD56^dim^ NK cells stimulated with LFL in all studied groups, presented using a nonlinear regression model (*n* = 27). (**F**) Comparison of CD107a expression in CD57^+^ and CD57^−^ CD56^dim^ NK cell subsets in samples stimulated with LFL in hCMV^high^ group (*n* = 16). (**G**) Comparison of HLA-DR expression by NKG2C^+^ and NKG2C^−^ CD56^dim^ NK cells in samples with stimulated LFL in hCMV^high^ group (*n* = 16). (**H**) Comparison of HLA-DR expression in CD57^+^ and CD57^−^ CD56^dim^ NK cell subsets in samples stimulated with LFL in hCMV^high^ group (*n* = 16). The colors of the symbols correspond to the groups, wherein green is hCMV^low^ and red is hCMV^high^. The colors of the boxes correspond to stimulation conditions, wherein gray—no stimulation and light yellow—stimulation with the LFL peptide (VMAPRTLFL). Data are presented as mean values with lines connecting symbols for each donor. Each symbol represents the mean of two replicates. The paired Wilcoxon statistical test was used to analyze the data. * *p* < 0.05, ** *p* < 0.01, and *** *p* < 0.001.

To examine whether the LFL peptide presented by autologous cells induces NK cell degranulation, CD3^neg^PBMC samples were stimulated with LFL for 16 h without exogenous cytokines. The expression levels of CD107a and HLA-DR were detected in both CD56^bright^ and CD56^dim^ NK cells (Figure 4A,C); therefore, to minimize the effect in less differentiated NK cells, we determined the degranulation and activation levels in CD56^dim^ NK cells alone. The gating strategy is depicted in Figure 1C. CD56^dim^ NK cells from the hCMV^high^ group exhibited a statistically significant increase in the level of degranulation in response to the LFL peptide (Figure 4A,B). CD56^dim^ NK cells from the hCMV^low^ group incubated with LFL showed no significant changes in the level of degranulation compared to unstimulated controls (Figure 4A,B). In addition to functional tests, we analyzed changes in the proportion of HLA-DR^+^ after LFL stimulation in the CD56^dim^ NK cell subpopulation. HLA-DR surface expression in CD56^dim^ NK cells is usually associated with their activation [50]. NK cells from the hCMV^high^ group were activated to a greater extent in samples interacting with the peptide compared to the control while the CD56^dim^ NK cell analysis of the hCMV^low^ group revealed no increase in HLA-DR expression after incubation with the LFL peptide (Figure 4C,D). Thus, the addition of the LFL peptide to the autologous cell system resulted in the degranulation and activation of CD56^dim^ NK cells of donors with moderate and high levels of hCMV-specific IgG.

No relationships between the proportion of CD107^+^ CD56^dim^ NK cells and percentages of NK cells expressing NKG2C, CD57, or KIR2DL2/DL3 were found (Supplementary Figure S4A), and a positive correlation between HLA-DR^+^ and KIR2DL2/DL3^+^ NK cell proportions was observed only in the hCMV^low^ group (Figure 4E). No associations were found for the proportions of HLA-DR^+^ cells and NKG2C^+^ or CD57^+^ NK cells (Supplementary Figure S4B).

Using multicolor flow cytometry, we have estimated degranulation in response to the LFL peptide in several CD56^dim^ NK cell subsets. In contrast to the IFNγ production assay, in the degranulation test, the CD57^−^ CD56^dim^ NK cells were more functionally active and displayed higher degranulation levels compared to CD57^+^ CD56^dim^ NK cells (Figure 4F). Interestingly, the percentages of CD107a^+^ NK cells were similar between NKG2C^+^ and NKG2C^−^ NK cells as well as between KIR2DL2/DL3^+^ and KIR2DL2/DL3^−^ cells in the CD56^dim^ NK cell subpopulation, and especially in the NKG2C^+^CD57^+^ subset (Supplementary Figure S4C–F), while the activation level (percentage of HLA-DR^+^ cells), similar to the IFNγ production level, was higher in NKG2C^+^ NK cells than in NKG2C^−^ NK cells in hCMV^high^ donors (Figure 4G, Supplementary Figure S4G). The CD57^−^ NK cells were more activated than the CD57^+^ NK cells (Figure 4H). The HLA-DR^+^ NK cell proportions were similar in KIR2DL2/DL3^+^ and KIR2DL2/DL3^−^ cells in the CD56^dim^ subpopulation and within the adaptive-like NKG2C^+^CD57^+^ cell subset (Supplementary Figure S4H,I). These findings highlighted that mostly less-differentiated CD57^−^ NK cells degranulated in response to the VMAPRTLFL peptide presented by autologous cells in vitro.

### 3.5. Cytokine Production by CD3^neg^PBMC Influenced by the LFL Peptide in hCMV^high^ Donors

In the previous sections, we have shown that incubation with the LFL peptide for 6 h resulted in the induction of IFNγ production in the NKG2C^+^CD57^+^KIR2DL2/DL3^+^ cell subset while the incubation for 16 h resulted in degranulation activity in CD57^−^ NK cells. To further investigate the phenomenon of the LFL-induced NK cell activation, supernatants of CD3^neg^PBMC cultures were collected after overnight incubation with LFL and concentrations of cytokines accumulated in the supernatants were measured. For this purpose, we randomly chose fifteen donors (nine from the hCMV^high^ group and six from the hCMV^low^ group). Only six out of twelve analyzed cytokines were detected in the supernatants. The concentrations of IFNa, IL-2, IL-4, IL-5, IL-9, and IL-17A, if present, were below the measurable threshold in all tested samples.

**Figure 5:**
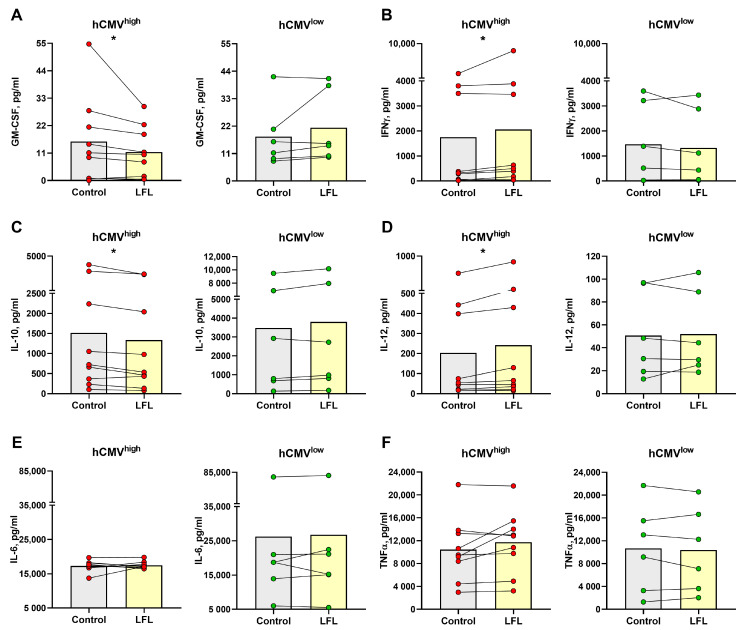
Concentration of cytokines in supernatants of CD3^neg^PBMC samples after the overnight cultivation. Concentration of (**A**) GM-CSF, (**B**) IFNγ, (**C**) IL-10, (**D**) IL-12, (**E**) IL-6, and (**F**) TNF-α in samples from hCMV^high^ (*n* = 9) and hCMV^low^ (*n* = 6) donors without stimulation (control) and with the LFL peptide stimulation (LFL). The colors of the symbols correspond to the groups, wherein green is hCMV^low^ and red is hCMV^high^. The colors of the boxes correspond to stimulation conditions, wherein gray—no stimulation and light yellow—stimulation with the LFL peptide (VMAPRTLFL). Data are presented as mean values with lines connecting symbols for each donor. Each symbol represents the mean of two replicates. The paired Wilcoxon statistical test was used to analyze the data. * *p* < 0.05.

The incubation with LFL without any exogenous supplementation resulted in a decrease in the GM-CSF level in culture supernatants only in samples from hCMV^high^ donors (Figure 5A). IFNγ supernatant levels increased in the hCMV^high^ donor group after the stimulation with LFL (Figure 5B), which corresponded to the results of Section 2. In the hCMV^high^ donor group, the concentration of anti-inflammatory cytokine IL-10 was lower in cell cultures incubated with the LFL peptide than in control samples (Figure 5C). In contrast, proinflammatory cytokine IL-12 was accumulated in response to the LFL peptide in hCMV^high^ donors (Figure 5). The CD3^neg^PBMC samples of hCMV^high^ donors exhibited highly variable IL-6 and TNF-α levels, and no significant difference in the production of these cytokines between control and LFL-treated samples was observed (Figure 5E,F). In the hCMV^low^ donor group, no statistically significant changes in NK-cell cytokine production responses to the LFL stimulation were observed for all cytokines tested (Figure 5).

### 3.6. NK Cells of hCMV^high^ Donors Proliferate Better in Response to the LFL Peptide and Subsequently form Distinct Antigen-Specific Subset

**Figure 6:**
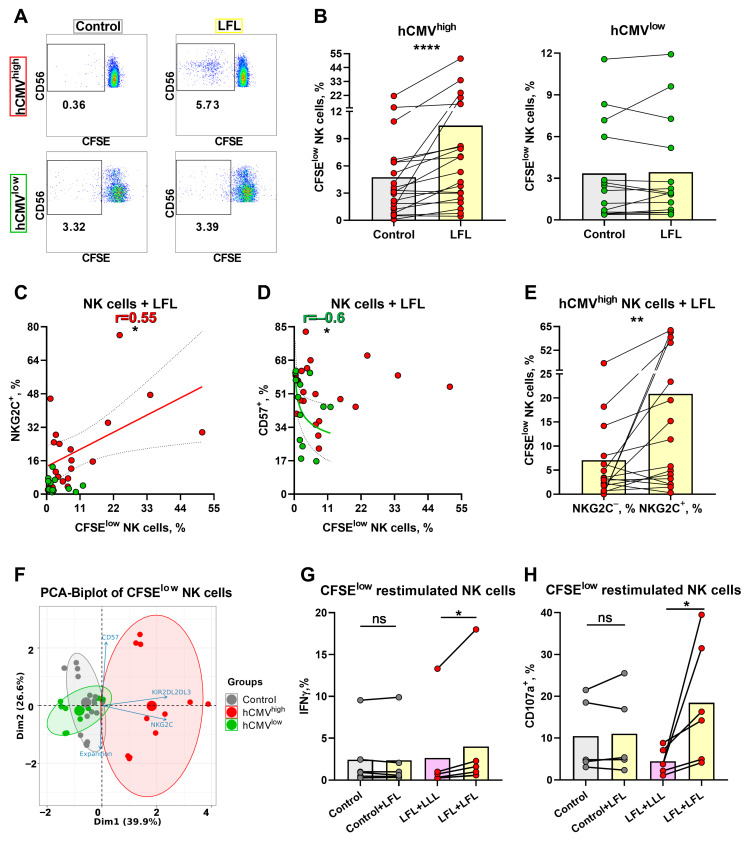
Proliferative potential of NK cells after 7 days of incubation with the LFL peptide and following characterization of CFSE^low^ NK cells. (**A**) Representative cytometric data on the percentages of CFSE^low^ NK cells in samples of one hCMV^high^ and one hCMV^low^ donor without stimulation and after stimulation with LFL. (**B**) Comparison of the proportions of CFSE^low^ NK cells in control and samples stimulated with LFL in hCMV^high^ (*n* = 20) and hCMV^low^ (*n* = 14) donors. (**C**) Spearman correlation analysis of NKG2C expression and the percentage of CFSE^low^ NK cells stimulated with LFL in all studied groups, presented using a nonlinear regression model (*n* = 34). (**D**) Spearman correlation analysis of CD57 expression and the percentage of CFSE^low^ NK cells stimulated with LFL in all studied groups, presented using a nonlinear regression model (*n* = 34). (**E**) Comparison of the percentage of CFSE^low^ NK cells in NKG2C^+^ and NKG2C^−^ NK cell subsets in samples stimulated with LFL in hCMV^high^ group (*n* = 16). (**F**) Principal component biplot showing relationship among grown CFSE^low^ NK cells from 9 donors (6 hCMV^high^ and 3 hCMV^low^, presented as grown from previous stimulation with hCMV (red and green, respectively) and previously unstimulated (control—gray)) and their phenotype characteristics (NKG2C, CD57, and KIR2DL2/DL3 percentages) and expansion rates. (**G**) Comparison of IFNγ production in grown CFSE^low^ NK cells in previously LFL-stimulated and control cells restimulated with LFL in 6 hCMV^high^ donors. (**H**) Comparison of the level of degranulation in grown CFSE^low^ NK cells in previously LFL-stimulated and control cells restimulated with LFL in 6 hCMV^high^ donors. The colors of the symbols correspond to the groups, wherein green is hCMV^low^ and red is hCMV^high^. The colors of the boxes correspond to stimulation conditions, wherein gray—no stimulation and light yellow—stimulation with the LFL peptide (VMAPRTLFL); light purple—stimulation with LLL peptide (VMAPQSLLL). Data are presented as mean values with lines connecting symbols for each donor. Each symbol represents the mean of two replicates. The paired Wilcoxon statistical test was used to analyze the data. Ns *p* > 0.05, * *p* < 0.05, ** *p* < 0.01, and **** *p* < 0.0001.

To further analyze the NK-cell response to LFL in a prolonged period of cultivation, the proliferative potential of NK cells was assessed using the detection of CFSE^low^ NK cells after a 7-day incubation with LFL. Since, in the previous section, we demonstrated that the NK cells were able to respond to the peptide without additional exogenous stimulation, for the proliferation test, we decided to not add any cytokines, either. The gating scheme is shown in Figure 1C. NK cells from hCMV^high^ donors stimulated with the LFL peptide showed an increase in the proportion of CFSE^low^ cells compared to the cells that were unstimulated (Figure 6A,B). In contrast, NK cells from hCMV^low^ donors had no differences in proliferation between cells cultured in the presence of the LFL peptide and control cells (Figure 6A,B). The analysis of the association between NKG2C expression in NK cells ex vivo and their proliferation rate in response to the LFL peptide showed a slight positive correlation for the hCMV^high^ group (Figure 6C). Additionally, a negative correlation was identified between CD57 level and percentage of CFSE^low^ NK cells for hCMV^low^ donors while no such dependence was demonstrated for hCMV^high^ donors (Figure 6D). There was no association between the proliferation of NK cells in response to the LFL peptide and KIR2DL2/DL3^+^ NK cells (Supplementary Figure S5A). A higher proportion of proliferating NK cells was found in the fraction of NKG2C^+^ cells compared to the NKG2C^−^ NK cells (Figure 6E). No statistically significant differences in the proliferation potential in response to LFL were identified between KIR2DL2/DL3^+^, CD57^+^, and their negative counterparts (Supplementary Figure S5B,C). Thus, the LFL peptide stimulated the proliferation of NK cells in individuals with moderate to high serum levels of hCMV IgG.

To study the characteristics of these highly proliferated NK cells in more detail, nine donors were chosen, six of whom had high and medium hCMV IgG titers (hCMV^high^ group), and three donors were from the hCMV^low^ group. Intensively proliferating in the presence of the LFL peptide and proliferating without stimulation (control), CD56^+^CD3^−^SYTOX^−^ CFSE pre-treated cells were isolated after 7 days using a cell sorter and then grown in microcultures (100 cells) with IL-2 and K562-mbIL21 feeder cells. Different expansion rates were observed in the microcultures 9–10 days after sorting. These data along with surface NKG2C, CD57, and KIR2DL2/DL3 expression values measured in these samples were used for the construction of the principal component analysis (PCA). There was identified a cluster of peptide-stimulated NK cells formed strictly from the hCMV^high^ donor data while control samples from the same donors, together with control and LFL-stimulated cells from hCMV^low^ donors, clustered separately from LFL-stimulated samples of hCMV^high^ donors (Figure 6F). This clusterization pattern supports the similarities of the hCMV-specific clonal NK cell populations obtained from different donors. To confirm their antigen-specific nature, these NK cells were further restimulated with LFL or with the control peptide using thawed autologous monocytes as antigen-presenting cells. The restimulation with the LFL peptide led to an increase in IFNγ production only in cultures grown from hCMV^high^ donor samples previously stimulated with this peptide (Figure 6G). NK cell degranulation measurement displayed similar results: NK cell degranulation potential after the restimulation with LFL was higher compared to the LFL-stimulated NK cells that had not been stimulated previously (control + LFL) (Figure 6H).

### 3.7. Transcriptome Analysis of CFSE^low^ NK Cells from hCMV^high^ Donor Revealed the Traits of Adaptive-like Cells in LFL-Stimulated Cultures

**Figure 7:**
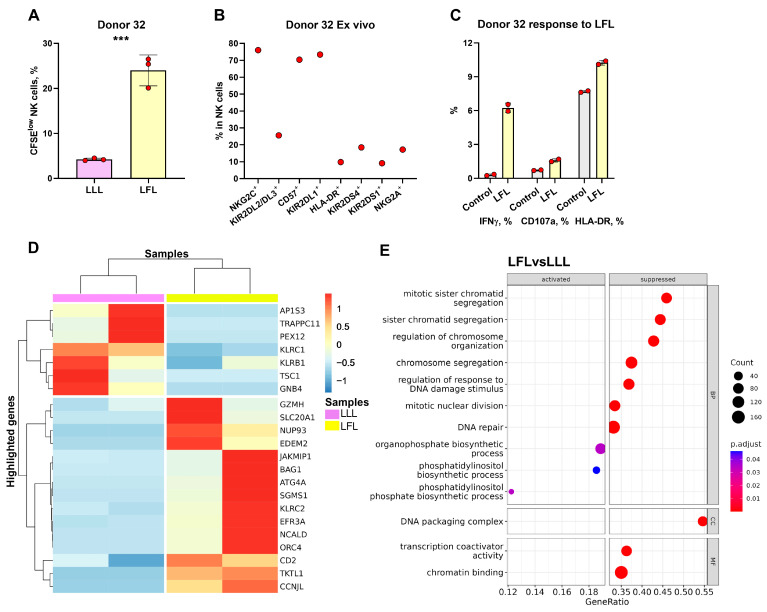
Transcriptional analysis of CFSE^low^ NK cells stimulated with LFL and LLL peptides. (**A**) Comparison of the proportions of CFSE^low^ NK cells in samples stimulated with LFL and LLL peptides from Donor 32. (**B**) Ex Vivo percentages of surface markers in NK cells of Donor 32. (**C**) Demonstration of the IFNγ production and CD107a and HLA-DR percentages after stimulation with LFL and LLL peptides. The colors of the boxes correspond to stimulation conditions, wherein gray—no stimulation, light yellow—stimulation with the LFL peptide (VMAPRTLFL), and light purple—stimulation with LLL peptide (VMAPQSLLL). Data are presented as means ± SDs with symbols representing the one replicate. The paired-T statistical test was used to analyze the data. *** *p* < 0.001. (**D**) Unsupervised hierarchical clustering of differentially expressed genes (LFC > 0.58, *p*-adjusted < 0.1): each column represents the samples of CFSE^low^ NK cells (stimulated with LFL (light yellow) and LLL (light purple)) from donor 32 and each row represents a gene. The heatmap indicates the level of gene expression: red indicated increased expression and blue indicates decreased expression. (**E**) Dotplot of GeneOntology gene set enrichment analysis of CFSE^low^ NK cells stimulated with LFL vs. LLL, where y-axis represents GO molecular pathways (Biological Processes (BP) Molecular Functions (MFs)), separated by sign and x-axis gene ratio. The greater the size of a circle is, the greater the number of genes involved in a pathway is, and the circles are colored based on *p*-adjusted values.

To further explore NK cells responding to the LFL peptide, we chose one donor to perform the RNA sequencing (RNAseq) of highly proliferating NK cells. Donor 32 exhibited the greatest difference in proliferation rate in response to the LFL peptide (Figure 7A). Moreover, this donor had an extremely high percentage of NKG2C^+^ NK cells and showed a significant increase in IFNγ production, degranulation, and activation levels in response to LFL (Figure 7B,C). Thus, the transcriptome of CFSE^low^ NK cells from this donor was studied. Due to technical reasons, only two replicates proceeded to differential expression analysis.

Through the unbiased analyses of differentially expressed genes (DEGs) and gene ontology (GO) network pathways, we identified that CFSE^low^ NK cells stimulated with the LFL peptide were characterized as adaptive cells with increased levels of metabolic activity (Figure 7D,E, Supplementary Figure S6). The upregulated genes in CFSE^low^ LFL-stimulated NK cells, compared to the NK cells stimulated with the LLL peptide, included genes encoding activating receptors (*KLRC2, CD2*); adaptive NK cell gene (*JAKMIP1*); gene encoding component of cytotoxic granules (*GZMH*); genes involved in mitosis (*CCNJL*), replication, and transcription (*ORC4*, *NUP93*); anti-apoptotic gene (*BAG1*); and genes involved in metabolism (*TKTL1*, *NCALD*, *EFR3A*, *SGMS1*, *ATG4A*, *EDEM2*, *SLC20A1*) (Figure 7D). Downregulated genes included genes encoding inhibitory receptors (*KLRC1*, *KLRB1*), genes involved in protein transport (*AP1S3*, *TRAPPC11*, *PEX12*), and genes involved in signaling processes (*FCER1G*, *TSC1*, *GNB4*) (Figure 7D).

## 4. Discussion

HCMV infection contributes to the accumulation of memory NKG2C^+^ NK cells [23,47,51]. The VMAPRTLFL (LFL) peptide presented in HLA-E was shown in studies to induce the strongest response of NKG2C^+^ NK cells [12,13]. Most of the NKG2C^+^ NK cell studies occurred under stimulation by different cytokines [13,41,52]. It has been shown that a combination of IL-15, IL-12, and IL-18 generates the development of cytokine-induced memory-like NK cells [53,54,55]. In this work, we avoided additional stimulation with exogenous cytokines to exclude the effect of facilitating the response of NK cells through co-activation. Unlike the previously used approaches, we analyzed the NK-cell response to the autologous presentation of the hCMV peptide (LFL) in the absence of T cells. The division of donors into two groups according to clusterization data based on the IgG titer to hCMV and markers associated with hCMV (NKG2C, CD57, KIR2DL2/DL3, HLA-DR) allowed us to investigate NK-cell reaction to the LFL peptide depending on not only the hCMV status but mostly on the equal contribution of these parameters. Moreover, using this approach, we were able to include, in the clusterization, an NKG2C null donor. The existence of such individuals (KLRC1^del/del^) in approximately 4% of the whole population has been observed in various countries [56,57]. On the other hand, it was shown recently that NK cells of NKG2C null donors may differentiate similarly with and without NKG2C, at least in the case of HIV co-infection [58]. A compensatory role of CD2 in the process of NK cell adaptation to hCMV infection was suggested in that study [58].

NKG2C^+^ NK cells were characterized previously by the increased production of IFNγ in response to contact interactions with target cells [59]. Hammer et al. demonstrated that NKG2C^+^ NK cells of hCMV^+^ individuals recognize and respond to LFL presented by RMA-S–HLA-E [13]. In this work, we have shown that NK cells in CD3^neg^PBMC samples from hCMV^high^ donors responded to the LFL peptide by producing an increased level of IFNγ while, under the same conditions, NK cells of hCMV^low^ donors did not show any changes in IFNγ production. The following analysis of NKG2C surface expression revealed that stimulation by the LFL peptide leads to a decrease in NKG2C expression in NK cells from both hCMV^high^ and hCMV^low^ donors. Perhaps, after NKG2C binding to the HLA-E/LFL complex on the surfaces of antigen-presenting cells, the NKG2C receptor internalized [52] or became less accessible to the monoclonal anti-NKG2C antibody. Presumably, NKG2C^+^ NK cells of hCMV^low^ donors also interacted with the LFL peptide; however, such interactions did not result in their activation and significant IFNγ production. Taking together, those data suggest that without exogenous cytokine stimulation, only hCMV-infection-experienced NK cells respond to the LFL peptide.

The analysis of the signaling pathways, the activation of which caused IFNγ production by LFL-stimulated NK cells, was conducted via the blocking of signaling molecules involved in the immune response of NK cells. DAP12 is known to be involved in the signal transduction of a range of activating NK-cell receptors including NKG2C [31]. The interaction of NKG2C with HLA-E results in the phosphorylation of ITAM motifs of DAP12, followed by the activation of SYK family molecules and then either the activation of the PI3K pathway or signaling through the Ras/Raf/Mek pathway [60]. Both of those pathways activate ERK1/2 [61,62]. The ability of those pathways to regulate the cytotoxic activity of NK cells has been clearly stated [61], but it is difficult to find data supporting the direct involvement of ERK1/2 in NK-cell IFNγ production after DAP12 activation. Those of our results obtained on the inhibition of PI3K also showed a decrease in IFNγ production, but not as strong as with the inhibition of ERK1/2. Possibly, the activation of ERK1/2 by the Ras/Raf/Mek pathway slightly compensated for the blocking of PI3K. Another way of induction of IFNγ production in NK cells is connected with the activation of the JAK/STAT pathway, mediated by cytokines such as IL-12, IL-21, and I-type IFNs [28,63]. However, the addition of the STAT1 inhibitor did not result in a decrease in IFNγ production in our experiments. Moreover, we have demonstrated that STAT3 inhibitors had no influence on IFNγ production. Considering that the activations of JAK2/TYK2/STAT1/2/3/4 and JAK1/3/STAT1/3 signaling from IL12R and IL21R, respectively result in IFNγ production [28,64,65], the lack of an inhibitory effect of fludorabine and cryptotanshinone may indicate that IFNγ production occurs indeed in response to peptides rather than cytokines. The autologous system without the addition of exogenous cytokines allowed us to identify the direct involvement of ERK1/2 and PI3K in the IFNγ production by NK cells in response to NKG2C activation induced by the LFL peptide.

NK cell degranulation is often measured upon incubation with target cell lines such as K562 [66,67]. On the other hand, in recent studies, the expression of CD107a was detected in response to peptides presented by dendritic cells or cell lines expressing specific HLA molecules [13,68]. NK cell degranulation induced by interaction with LFL-loaded HLA-E on the surfaces of autologous cells may presumably lead to the elimination of cells presenting a pathogen pattern (hCMV) or too much self (HLA-G). Here, similar to IFNγ production, the degranulation of CD56^dim^ NK cells increased in response to the LFL peptide only in samples of hCMV^high^ donors, which additionally supports the notion that NK cell pre-experience with hCMV infection is needed to perform peptide-specific degranulation. Simultaneous analysis of HLA-DR expression further confirmed the activation of CD56^dim^ NK cells from hCMV^high^ donors stimulated with LFL.

Since we conducted the experiments without exogenous cytokines, the endogenous cytokines might be still produced by autologous monocytes and dendritic cells and need to be described. In this work, we demonstrated the enlargement of IFNγ in supernatants of hCMV^high^ donors stimulated with the LFL peptide, which corresponded to the results of intracellular IFNγ measurements. The increased IL-12 produced by antigen-presenting cells after the incubation with LFL may stimulate the expansion of NKG2C^+^ NK cells [39]. However, the IL-12 concentration usually applied for exogenous addition in various studies ranges around 10 ng/mL [13,40], which is 100 times more than that detected in supernatants of most of the analyzed donors. Possibly, the minor amounts of IL-12 produced by autologous cells in our system promote the NK-cell response but do not induce the increase in NKG2C^+^ NK cells. Along with the elevated level of IL-12, we have observed a decrease in the anti-inflammatory cytokine IL-10 level, which is produced by different types of immune cells [69]. Taking into consideration other observed shifts in cytokine concentrations, our results suggest slight changes towards the proinflammatory environment in response to the LFL peptide. Previous studies reported upregulated TNF-α production by hCMV-specific NK cells [13,70]; however, in our model, changes both in TNF-α as well as in IL-6 concentrations were dependent on the donors and did not cause any significant shifts. Moreover, we would like to highlight that the LFL peptide did not affect any of the measured cytokine levels in samples from hCMV^low^ donors. Overall, the effects of cytokines are cooperative and depend on their combinations, doses, and the state of a responding cell.

Interestingly, NK cells lacking or having low titers of hCMV-specific IgG, despite the presence of NKG2C, did not increase IFNγ production and degranulation in response to the peptide whereas previously, it was shown that the NKG2C^+^ subpopulation is able to respond with intense proliferation to the HLA-E-presented peptide [13]. IL-12 production by antigen-presenting cells may be accompanied by the de novo induction of NKG2A expression by NK cells, which may inhibit the HLA-E-induced response in seronegative or low-seropositive donors. The NKG2A expression is expected to be triggered more easily in less differentiated cells.

Proliferation in response to peptides is one of the main signs of antigen-specific cytotoxic T cells and can be measured through CFSE staining [71,72]. In our work, we applied such a technique to identify the LFL-responding NK cells. NK cells of highly seropositive donors actively proliferated in response to the LFL peptide while the addition of LFL did not affect the proliferation of NK cells from seronegative and low-IgG-hCMV donors. Further analysis of such CFSE^low^ NK cells revealed that those cells obtained from hCMV^high^ donors form a cluster distinct from control cells of the same donors and cells of hCMV^low^ donors according to expansion rate and surface marker expression (NKG2C, CD57, KIR2DL2/DL3). Those results confirm the similarity between hCMV-specific NK cells in different donors who experienced the hCMV infection.

NKG2C^+^ NK cells accumulated in hCMV infection were shown to express CD57 and KIR2DL2/DL3 [24]. In the analysis of the phenotype of responding to the LFL peptide cells, we demonstrated higher levels of IFNγ production in NKG2C^+^, CD57^+^, and KIR2DL2/DL3^+^ NK cells and KIR2DL2/DL3^+^ cell proportion in the NKG2C^+^CD57^+^ subset compared to their negative counterpart. However, other assays did not show such a dependency, or even the reverse association. The level of degranulation in response to the LFL peptide differed only in CD57^+^/^−^ NK cells with a predominance of CD107 expression in CD57^−^ NK cells. CD57^−^ NK cells were shown to perform higher degranulation activity than CD57^+^ cells [73,74]. Perhaps CD57^−^ cells spontaneously degranulate better not due to the binding to the peptide, but under the influence of cytokines produced by adaptive CD57^+^NKG2C^+^ cells, which exhibit decreased natural cytotoxicity but produce IFNγ that activates less differentiated cells. The level of NK cell activation measured via the percentage of HLA-DR^+^ cells was higher in NKG2C^+^ and CD57^−^ NK cells compared to NKG2C^−^ and CD57^+^ NK cells, respectively, corresponding to our previous study on the ex vivo phenotype of hCMV^+^ donors [25,50]. Interestingly, we demonstrated a correlation between KIR2DL2/DL3 and HLA-DR expression only in hCMV^low^ donors; furthermore, there was no association between KIR2DL2/DL3 level and other measured parameters (IFNγ production and proliferation). Indeed, a higher proliferation level of NKG2C^+^ NK cells, compared to NKG2C^−^ NK cells, was observed. Along with a positive correlation between NKG2C expression and the percentage of proliferation of NK cells in hCMV^high^ donors, those results indicate that NKG2C^+^ NK cells respond to LFL by increasing proliferation. A higher proliferation potential was detected in CD57^−^ NK cells compared to CD57^+^ cells. Previously, we showed that CD57^−^NKG2C^+^ NK cells proliferate better compared to CD57^+^NKG2C^+^ cells [26], and we demonstrated the ability of CD57^+^ NK cells to lose CD57 expression upon cultivation [26]. Therefore, we cannot exclude that the NKG2C^+^ NK cells responding to the LFL peptide may partially lose the expression of CD57 during activation.

Previous studies investigating hCMV-specific NK cells typically analyzed the differences in NKG2C^+^ and NKG2C^−^ NK cells [40,70] or the total subpopulation of NKG2C^+^ cells accumulated after stimulation with IL-12, IL-18, and different peptides [13]. In the present work, for the first time, we emphasize the main differences between proliferating NK cells in response to the LFL peptide and LLL peptide without additional exogenous stimulation by cytokines in an example of one donor. The transcriptomic profiling of CFSE^low^ NK cells responding to the LFL peptide revealed the traits of adaptive-like cells. The upregulation of the expression of *KLRC2* encoding the NKG2C receptor indeed indicates the higher proliferation of the NKG2C^+^ NK cells stimulated with LFL compared to those with LLL stimulation. The upregulation of *JAKMIP1* has also been found in antigen-experienced T cells [75], and recently, an increase in *JAKMIP1* was found in NKG2C^+^ NK cells compared to NKG2C^−^ NK cells in hCMV^+^ individuals [40]. The upregulation of CD2, an activating NK-cell receptor, and *GZMH*, encoding a component of cytotoxic granules, was also observed in NKG2C^+^ NK cells [13,40,71]. Furthermore, the essential role of CD2 expression in hCMV infection was demonstrated [76,77,78,79]. The expression of *TKTL1*, encoding the enzyme involved in the pentose phosphate pathway [80], was upregulated in tumor-induced memory NK cells [81]. Moreover, we identified several genes involved in various metabolic processes and genes regulating the cell cycle. The downregulated genes such as *KLRC1* and *KLRB1*, encoding inhibitory NK-cell receptors, were shown to be enriched in canonical NKG2C^−^ NK cells [13,40,70]. Another gene, *FCERG1*, which encodes the FcRγ adaptor protein for NK-cell-activating receptors CD16, NKp30, and NKp46, is also commonly downregulated in memory NK cells [82]. Hence, the gene expression patterns of CFSE^low^ LFL-specific NK cells obtained from one donor indicate a more activated adaptive-like state of cells compared to CFSE^low^ NK cells incubated with the LLL peptide.

Throughout our work, the influence of the LFL peptide was demonstrated only in samples from hCMV^high^ donors even though most of the hCMV^low^ donors also expressed NKG2C. Interestingly, the LFL sequence was determined approximately in 1–2% of hCMV strains [13,83] while the human HLA-G leader sequence contains LFL [83]. In healthy conditions, the level of HLA-G expression is restricted to immune-privileged tissues [84,85]; however, during various viral infections, the upregulation of HLA-G is observed [86,87,88,89]. Apparently, the presence of hCMV infection, which generates different HLA-E-stabilizing peptides, induces the formation of hCMV-specific NK cells, the response of which is accelerated then by the presentation of the LFL peptide derived from HLA-G.

## 5. Conclusions

Taken together, the VMAPRTLFL peptide, presented by autologous cells, in the absence of T cells and exogenous cytokines, causes the activation of NK cells of individuals possessing moderate to high levels of IgG to hCMV. This activation is characterized by elevated IFNγ production, increased degranulation levels, a higher proportion of HLA-DR^+^ cells, and enhanced proliferation. The induction of IFNγ occurs through the activation of PI3K and Ras/Raf/Mek pathways, inducing the activation of ERK1/2 and not via endogenous cytokine. The cytokines created in CD3^neg^PBMC samples of hCMV^high^ donors shift the cell reactions to the proinflammatory state. Additionally, the proliferating NK cells, in response to the LFL peptide, form similar clonal populations in different hCMV^high^ donors in contrast to hCMV^low^ donors. The transcriptome of such highly proliferated NK cells in response to LFL differs from LLL-stimulated cells by the alterations in genes typical for adaptive-like NK cells and by metabolic activation. These data support the presence of hCMV-associated adaptive NK cells in individuals who have well-defined hCMV-specific B-cell responses.

## Data Availability

The datasets presented in this study can be found in online repositories. The names of the repository/repositories and accession number(s) can be found below: https://www.ncbi.nlm.nih.gov/bioproject/1056140, assessed on 23 December 2023.

## Supplementary Materials

The following supporting information can be downloaded at: https://www.mdpi.com/article/10.3390/cells13060530/s1. Supplementary Figure S1. Representative cytometric data of HLA-E surface expression without and after stimulation with LLL/LFL in NK cells. Supplementary Figure S2. IFNγ production by NK cells upon stimulation with the LFL peptide. (A) Comparison of the proportions of CD56^bright^ and CD56^dim^ NK cells producing IFNγ (*n* = 34). (B) Spearman correlation analysis of CD57 expression and IFNγ production by NK cells stimulated with LFL in all studied groups, presented using a nonlinear regression model (*n* = 34). (C) Spearman correlation analysis of KIR2DL2/DL3 expression and IFNγ production by NK cells stimulated with LFL in all studied groups (*n* = 34). (D) Comparison of IFNγ production in NKG2C^+^ and NKG2C^−^ NK cells in samples stimulated with LFL in the hCMV^low^ group (*n* = 13). (E) Fold change of IFNγ production by CD57^+^ and KIR2DDL2DL3^+^ NK cells, separated into two subgroups (subgroup 1—*n* = 12, subgroup 2—*n* = 8 of hCMV^high^ donors). (F) The percentage of NKG2C+, CD57+, KIR2DL2/DL3^+^ NK cells in two subgroups, identified in (C) (subgroup 1—*n* = 12, subgroup 2—*n* = 8 of hCMV^high^ donors). The color of the symbols corresponds to the groups, where green is hCMV^low^, red is hCMV^high^. NK cells were stimulated with the LFL peptide (VMAPRTLFL). Data are presented as symbols representing the mean of two replicates (B,C), mean values with lines connecting symbols for each donor (D,E), mean ± SD (A,F). The paired Wilcoxon test was used to analyze the data (D,E), and the T test was used for unpaired data (A,F). * *p* < 0.05, ** *p* < 0.01, *** *p* < 0.001. Supplementary Figure S3. Effect of simultaneous addition of STAT1 (Fludorabine) and STAT3 (Cryptotanshinone) inhibitors on the level of IFNγ production in NK cells of hCMV^high^ donors (*n* = 6). Data are presented as mean values with lines connecting symbols for each donor. Each symbol represents the mean of two replicates. The paired Friedman statistical test was used to analyze the data. * *p* < 0.05, ** *p* < 0.01. Supplementary Figure S4. Degranulation and activation of NK cells upon stimulation with the LFL peptide. (A) Spearman correlation analysis of NKG2C, CD57, KIR2DL2/DL3 expression and level of CD107a expression by NK cells stimulated with LFL in all studied groups (*n* = 27). (B) Spearman correlation analysis of NKG2C, CD57 expression and level of HLA-DR expression by NK cells stimulated with LFL in all studied groups (*n* = 27). (C) Comparison of CD107a expression by NKG2C^+^ and NKG2C^−^ NK cells in samples stimulated with LFL in hCMV^high^ group (*n* = 16). (D) Comparison of CD107a expression by NKG2C^+^ and NKG2C^−^ NK cells in samples stimulated with LFL in hCMV^low^ group (*n* = 11). (E) Comparison of CD107a expression by KIR2DL2/DL3^+^ and KIR2DL2/DL3^−^ NK cells in samples stimulated with LFL in hCMV^high^ group (*n* = 16). (F) Comparison of CD107a expression by KIR2DL2/DL3^+^ and KIR2DL2/DL3^−^ NKG2C+CD57+NK cells in samples stimulated with LFL in hCMV^high^ group (*n* = 16). (G) Comparison of HLA-DR expression by NKG2C^+^ and NKG2C^−^ NK cells in samples stimulated with LFL in hCMV^low^ group (*n* = 11). (H) Comparison of HLA-DR expression by KIR2DL2/DL3^+^ and KIR2DL2/DL3^−^ NK cells in samples with stimulated LFL in hCMV^high^ group (*n* = 16). (I) Comparison of HLA-DR expression by KIR2DL2/DL3^+^ and KIR2DL2/DL3^−^ NKG2C^+^CD57^+^NK cells in samples stimulated with LFL in hCMV^high^ group (*n* = 16). The color of the symbols corresponds to the groups, wherein green—hCMV^low^, red—hCMV^high^. The color of the boxes corresponds to stimulation conditions, where gray is no stimulation and light yellow—stimulation with the LFL peptide (VMAPRTLFL). Data are presented as mean values with lines connecting symbols for each donor. Each symbol represents the mean of two replicates. Supplementary Figure S5. Proliferative potential of NK cells after 7 days of incubation with the LFL peptide, and following characterization of CFSE^low^ NK cells. (A) Spearman correlation analysis of KIR2DL2/DL3 expression and the percentage of CFSE^low^ NK cells stimulated with LFL in all studied groups (*n* = 34). (B) Comparison of the percentage of CFSE^low^ NK cells in KIR2DL2/DL3^+^ and KIR2DL2/DL3^−^ NK cells in samples stimulated with LFL in hCMV^high^ group (*n* = 16). (C) Comparison of the percentage of CFSE^low^ NK cells in CD57^+^ and CD57^−^ NK cells in samples stimulated with LFL in hCMV^high^ group (*n* = 3). The color of the symbols corresponds to the groups, where green is hCMV^low^, red is hCMV^high^. The color of the boxes corresponds to stimulation conditions, wherein gray—no stimulation and light yellow—stimulation with the LFL peptide (VMAPRTLFL). Data are presented as mean values with lines connecting symbols for each donor. Each symbol represents the mean of two replicates. Supplementary Figure S6. Volcano plot comparing LFL-stimulated with LLL-stimulated CFSE^low^ NK cells significant 96 out of 130 up-regulated and down-regulated.

## References

[1] Sivori S., Vacca P., Del Zotto G., Munari E., Mingari M.C., Moretta L. (2019). Human NK Cells: Surface Receptors, Inhibitory Checkpoints, and Translational Applications. Cell. Mol. Immunol..

[2] Paust S., Von Andrian U.H. (2011). Natural Killer Cell Memory. Nat. Immunol..

[3] Zuhair M., Smit G.S.A., Wallis G., Jabbar F., Smith C., Devleesschauwer B., Griffiths P. (2019). Estimation of the Worldwide Seroprevalence of Cytomegalovirus: A Systematic Review and Meta-Analysis. Rev. Med. Virol..

[4] Jackson S.E., Mason G.M., Wills M.R. (2011). Human Cytomegalovirus Immunity and Immune Evasion. Virus Res..

[5] Tomasec P., Braud V.M., Rickards C., Powell M.B., McSharry B.P., Gadola S., Cerundolo V., Borysiewicz L.K., McMichae A.J., Wilkinson G.W.G. (2000). Surface Expression of HLA-E, an Inhibitor of Natural Killer Cells, Enhanced by Human Cytomegalovirus GpUL40. Science.

[6] Barnes P.D., Grundy J.E. (1992). Down-Regulation of the Class I HLA Heterodimer and Beta 2-Microglobulin on the Surface of Cells Infected with Cytomegalovirus. J. Gen. Virol..

[7] Halenius A., Gerke C., Hengel H. (2014). Classical and Non-Classical MHC I Molecule Manipulation by Human Cytomegalovirus: So Many Targets—But How Many Arrows in the Quiver?. Cell. Mol. Immunol..

[8] Warren A.P., Ducroq D.H., Lehner P.J., Borysiewicz L.K. (1994). Human Cytomegalovirus-Infected Cells Have Unstable Assembly of Major Histocompatibility Complex Class I Complexes and Are Resistant to Lysis by Cytotoxic T Lymphocytes. J. Virol..

[9] Tomazin R., Boname J., Hedge N.R., Lewinsohn D.M., Altschuler Y., Jones T.R., Cresswell P., Nelson J.A., Riddell S.R., Johnson D.C. (1999). Cytomegalovirus US2 Destroys Two Components of the MHC Class II Pathway, Preventing Recognition by CD4+ T Cells. Nat. Med..

[10] Wang E.C.Y., McSharry B., Retiere C., Tomasec P., Williams S., Borysiewicz L.K., Braud V.M., Wilkinson G.W.G. (2002). From the Cover: UL40-Mediated NK Evasion during Productive Infection with Human Cytomegalovirus. Proc. Natl. Acad. Sci. USA.

[11] Ulbrecht M., Martinozzi S., Grzeschik M., Hengel H., Ellwart J.W., Pla M., Weiss E.H. (2000). Cutting Edge: The Human Cytomegalovirus UL40 Gene Product Contains a Ligand for HLA-E and Prevents NK Cell-Mediated Lysis. J. Immunol..

[12] Heatley S.L., Pietra G., Lin J., Widjaja J.M.L., Harpur C.M., Lester S., Rossjohn J., Szer J., Schwarer A., Bradstock K. (2013). Polymorphism in Human Cytomegalovirus UL40 Impacts on Recognition of Human Leukocyte Antigen-E (HLA-E) by Natural Killer Cells. J. Biol. Chem..

[13] Hammer Q., Rückert T., Borst E.M., Dunst J., Haubner A., Durek P., Heinrich F., Gasparoni G., Babic M., Tomic A. (2018). Peptide-Specific Recognition of Human Cytomegalovirus Strains Controls Adaptive Natural Killer Cells Article. Nat. Immunol..

[14] Sijmons S., Thys K., Ngwese M.M., Van Damme E., Dvorak J., Van Loock M., Li G., Tachezy R., Busson L., Aerssens J. (2015). High-Throughput Analysis of Human Cytomegalovirus Genome Diversity Highlights the Widespread Occurrence of Gene-Disrupting Mutations and Pervasive Recombination. J. Virol..

[15] Dargan D.J., Douglas E., Cunningham C., Jamieson F., Stanton R.J., Baluchova K., McSharry B.P., Tomasec P., Emery V.C., Percivalle E. (2010). Sequential Mutations Associated with Adaptation of Human Cytomegalovirus to Growth in Cell Culture. J. Gen. Virol..

[16] Cunningham C., Gatherer D., Hilfrich B., Baluchova K., Dargan D.J., Thomson M., Griffiths P.D., Wilkinson G.W.G., Schulz T.F., Davison A.J. (2010). Sequences of Complete Human Cytomegalovirus Genomes from Infected Cell Cultures and Clinical Specimens. J. Gen. Virol..

[17] Tomasec P., Wang E.C.Y., Davison A.J., Vojtesek B., Armstrong M., Griffin C., McSharry B.P., Morris R.J., Llewellyn-Lacey S., Rickards C. (2005). Downregulation of Natural Killer Cell-Activating Ligand CD155 by Human Cytomegalovirus UL141. Nat. Immunol..

[18] Davison A.J., Akter P., Cunningham C., Dolan A., Addison C., Dargan D.J., Hassan-Walker A.F., Emery V.C., Griffiths P.D., Wilkinson G.W.G. (2003). Homology between the Human Cytomegalovirus RL11 Gene Family and Human Adenovirus E3 Genes. J. Gen. Virol..

[19] Valés-Gómez M., Reyburn H.T., Erskine R.A., López-Botet M., Strominger J.L. (1999). Kinetics and Peptide Dependency of the Binding of the Inhibitory NK Receptor CD94/NKG2-A and the Activating Receptor CD94/NKG2-C to HLA-E. EMBO J..

[20] Kaiser B.K., Barahmand-pour F., Paulsene W., Medley S., Geraghty D.E., Strong R.K. (2005). Interactions between NKG2x Immunoreceptors and HLA-E Ligands Display Overlapping Affinities and Thermodynamics. J. Immunol..

[21] Huisman B.D., Guan N., Rückert T., Garner L., Singh N.K., McMichael A.J., Gillespie G.M., Romagnani C., Birnbaum M.E. (2023). High-Throughput Characterization of HLA-E-Presented CD94/NKG2x Ligands Reveals Peptides Which Modulate NK Cell Activation. Nat. Commun..

[22] Lodoen M.B., Lanier L.L. (2005). Viral Modulation of NK Cell Immunity. Nat. Rev. Microbiol..

[23] Gumá M., Budt M., Sáez A., Brckalo T., Hengel H., Angulo A., López-Botet M. (2006). Expansion of CD94/NKG2C+ NK Cells in Response to Human Cytomegalovirus-Infected Fibroblasts. Blood.

[24] Wu Z., Sinzger C., Frascaroli G., Reichel J., Bayer C., Wang L., Schirmbeck R., Mertens T. (2013). Human Cytomegalovirus-Induced NKG2Chi CD57hi Natural Killer Cells Are Effectors Dependent on Humoral Antiviral Immunity. J. Virol..

[25] Kovalenko E.I., Streltsova M.A., Kanevskiy L.M., Erokhina S.A., Telford W.G. (2017). Identification of Human Memory-Like NK Cells. Curr. Protoc. Cytom..

[26] Kobyzeva P.A., Streltsova M.A., Erokhina S.A., Kanevskiy L.M., Telford W.G., Sapozhnikov A.M., Kovalenko E.I. (2020). CD56dim CD57- NKG2C+ NK Cells Retaining Proliferative Potential Are Possible Precursors of CD57+ NKG2C+ Memory-like NK Cells. J. Leukoc. Biol..

[27] Béziat V., Liu L.L., Malmberg J.A., Ivarsson M.A., Sohlberg E., Björklund A.T., Retière C., Sverremark-Ekström E., Traherne J., Ljungman P. (2013). NK Cell Responses to Cytomegalovirus Infection Lead to Stable Imprints in the Human KIR Repertoire and Involve Activating KIRs. Blood.

[28] Gotthardt D., Trifinopoulos J., Sexl V., Putz E.M. (2019). JAK/STAT Cytokine Signaling at the Crossroad of NK Cell Development and Maturation. Front. Immunol..

[29] Freeman B.E., Raué H.-P., Hill A.B., Slifka M.K. (2015). Cytokine-Mediated Activation of NK Cells during Viral Infection. J. Virol..

[30] Sim M.J.W., Rajagopalan S., Altmann D.M., Boyton R.J., Sun P.D., Long E.O. (2019). Human NK Cell Receptor KIR2DS4 Detects a Conserved Bacterial Epitope Presented by HLA-C. Proc. Natl. Acad. Sci. USA.

[31] Lanier L.L., Corliss B., Wu J., Phillips J.H. (1998). Association of DAP12 with Activating CD94/NKG2C NK Cell Receptors. Immunity.

[32] Chen Y., Lu D., Churov A., Fu R. (2020). Research Progress on NK Cell Receptors and Their Signaling Pathways. Mediat. Inflamm..

[33] de Rham C., Ferrari-Lacraz S., Jendly S., Schneiter G., Dayer J.M., Villard J. (2007). The Proinflammatory Cytokines IL-2, IL-15 and IL-21 Modulate the Repertoire of Mature Human Natural Killer Cell Receptors. Arthritis Res. Ther..

[34] Terrén I., Orrantia A., Mosteiro A., Vitallé J., Zenarruzabeitia O., Borrego F. (2021). Metabolic Changes of Interleukin-12/15/18-Stimulated Human NK Cells. Sci. Rep..

[35] Lauwerys B.R., Renauld J.C., Houssiau F.A. (1999). Synergistic Proliferation and Activation of Natural Killer Cells by Interleukin 12 and Interleukin 18. Cytokine.

[36] Cheon S., Song S.B., Jung M., Park Y., Bang J.W., Kim T.S., Park H., Kim C.H., Yang Y.-h., Bang S.I. (2008). Sphingosine Kinase Inhibitor Suppresses IL-18-Induced Interferon-Gamma Production through Inhibition of P38 MAPK Activation in Human NK Cells. Biochem. Biophys. Res. Commun..

[37] Dybkaer K., Iqbal J., Zhou G., Geng H., Xiao L., Schmitz A., d’Amore F., Chan W.C. (2007). Genome Wide Transcriptional Analysis of Resting and IL2 Activated Human Natural Killer Cells: Gene Expression Signatures Indicative of Novel Molecular Signaling Pathways. BMC Genom..

[38] Caligiuri M.A. (2008). Human Natural Killer Cells. Blood.

[39] Rölle A., Pollmann J., Ewen E.M., Le V.T.K., Halenius A., Hengel H., Cerwenka A. (2014). IL-12–Producing Monocytes and HLA-E Control HCMV-Driven NK Cell Expansion. J. Clin. Investig..

[40] Rückert T., Lareau C.A., Mashreghi M.F., Ludwig L.S., Romagnani C. (2022). Clonal Expansion and Epigenetic Inheritance of Long-Lasting NK Cell Memory. Nat. Immunol..

[41] Rölle A., Meyer M., Calderazzo S., Jäger D., Momburg F. (2018). Distinct HLA-E Peptide Complexes Modify Antibody-Driven Effector Functions of Adaptive NK Cells. Cell Rep..

[42] Streltsova M., Erokhina S., Kanevskiy L., Grechikhina M., Kobyzeva P., Lee D., Telford W., Sapozhnikov A., Kovalenko E. (2019). Recurrent Stimulation of Natural Killer Cell Clones with K562 Expressing Membrane-Bound Interleukin-21 Affects Their Phenotype, Interferon-γ Production, and Lifespan. Int. J. Mol. Sci..

[43] Bray N.L., Pimentel H., Melsted P., Pachter L. (2016). Near-Optimal Probabilistic RNA-Seq Quantification. Nat. Biotechnol..

[44] Love M.I., Huber W., Anders S. (2014). Moderated Estimation of Fold Change and Dispersion for RNA-Seq Data with DESeq2. Genome Biol..

[45] Wu T., Hu E., Xu S., Chen M., Guo P., Dai Z., Feng T., Zhou L., Tang W., Zhan L. (2021). ClusterProfiler 4.0: A Universal Enrichment Tool for Interpreting Omics Data. Innovation.

[46] Sottile R., Panjwani M.K., Lau C.M., Daniyan A.F., Tanaka K., Barker J.N., Brentjens R.J., Sun J.C., Le Luduec J.B., Hsu K.C. (2021). Human Cytomegalovirus Expands a CD8+ T Cell Population with Loss of BCL11B Expression and Gain of NK Cell Identity. Sci. Immunol..

[47] Lopez-Vergès S., Milush J.M., Schwartz B.S., Pando M.J., Jarjoura J., York V.A., Houchins J.P., Miller S., Kang S.-M., Norris P.J. (2011). Expansion of a Unique CD57^+^NKG2Chi Natural Killer Cell Subset during Acute Human Cytomegalovirus Infection. Proc. Natl. Acad. Sci. USA.

[48] Hanafi L.A., Gauchat D., Godin-Ethier J., Possamaï D., Duvignaud J.B., Leclerc D., Grandvaux N., Lapointe R. (2014). Fludarabine Downregulates Indoleamine 2,3-Dioxygenase in Tumors via a Proteasome-Mediated Degradation Mechanism. PLoS ONE.

[49] Honda M., Kanno T., Fujita Y., Gotoh A., Nakano T., Nishizaki T. (2012). Mesothelioma Cell Proliferation through Autocrine Activation of PDGF-Ββ Receptor. Cell. Physiol. Biochem..

[50] Erokhina S.A., Streltsova M.A., Kanevskiy L.M., Telford W.G., Sapozhnikov A.M., Kovalenko E.I. (2018). HLA-DR+ NK Cells Are Mostly Characterized by Less Mature Phenotype and High Functional Activity. Immunol. Cell Biol..

[51] Gumá M., Angulo A., Vilches C., Gómez-Lozano N., Malats N., López-Botet M. (2004). Imprint of Human Cytomegalovirus Infection on the NK Cell Receptor Repertoire. Blood.

[52] Lauterbach N., Wieten L., Popeijus H.E., Voorter C.E.M., Tilanus M.G.J. (2015). HLA-E Regulates NKG2C+ Natural Killer Cell Function through Presentation of a Restricted Peptide Repertoire. Hum. Immunol..

[53] Sun J.C., Madera S., Bezman N.A., Beilke J.N., Kaplan M.H., Lanier L.L. (2012). Proinflammatory Cytokine Signaling Required for the Generation of Natural Killer Cell Memory. J. Exp. Med..

[54] Romee R., Schneider S.E., Leong J.W., Chase J.M., Keppel C.R., Sullivan R.P., Cooper M.A., Fehniger T.A. (2012). Cytokine Activation Induces Human Memory-like NK Cells. Blood.

[55] Cooper M.A., Elliott J.M., Keyel P.A., Yang L., Carrero J.A., Yokoyama W.M. (2009). Cytokine-Induced Memory-like Natural Killer Cells. Proc. Natl. Acad. Sci. USA.

[56] Goncalves A., Makalo P., Joof H., Burr S., Ramadhani A., Massae P., Malisa A., Mtuy T., Derrick T., Last A.R. (2016). Differential Frequency of NKG2C/KLRC2 Deletion in Distinct African Populations and Susceptibility to Trachoma: A New Method for Imputation of KLRC2 Genotypes from SNP Genotyping Data. Hum. Genet..

[57] Miyashita R., Tsuchiya N., Hikami K., Kuroki K., Fukazawa T., Bijl M., Kallenberg C.G.M., Hashimoto H., Yabe T., Tokunaga K. (2004). Molecular Genetic Analyses of Human NKG2C (KLRC2) Gene Deletion. Int. Immunol..

[58] Comeau E.M., Holder K.A., Fudge N.J., Grant M.D. (2019). Cytomegalovirus-Driven Adaption of Natural Killer Cells in NKG2Cnull Human Immunodeficiency Virus-Infected Individuals. Viruses.

[59] Luetke-Eversloh M., Hammer Q., Durek P., Nordström K., Gasparoni G., Pink M., Hamann A., Walter J., Chang H.D., Dong J. (2014). Human Cytomegalovirus Drives Epigenetic Imprinting of the IFNG Locus in NKG2Chi Natural Killer Cells. PLoS Pathog..

[60] Vivier E., Nunès J.A., Vély F. (2004). Natural Killer Cell Signaling Pathways. Science.

[61] Jiang K., Zhong B., Gilvary D.L., Corliss B.C., Hong-Geller E., Wei S., Djeu J.Y. (2000). Pivotal Role of Phosphoinositide-3 Kinase in Regulation of Cytotoxicity in Natural Killer Cells. Nat. Immunol..

[62] Crews C.M., Erikson R.L. (1992). Purification of a Murine Protein-Tyrosine/Threonine Kinase That Phosphorylates and Activates the Erk-1 Gene Product: Relationship to the Fission Yeast Byr1 Gene Product. Proc. Natl. Acad. Sci. USA.

[63] Lurie R.H., Platanias L.C. (2005). Mechanisms of Type-I- and Type-II-Interferon-Mediated Signalling. Nat. Rev. Immunol..

[64] Vivier E., Ugolini S., Nunès J.A. (2013). ADAPted Secretion of Cytokines in NK Cells. Nat. Immunol..

[65] Strobl B., Stoiber D., Sexl V., Mueller M. (2011). Tyrosine Kinase 2 (TYK2) in Cytokine Signalling and Host Immunity. Front. Biosci. (Landmark Ed.).

[66] Alter G., Malenfant J.M., Delabre R.M., Burgett N.C., Yu X.G., Lichterfeld M., Zaunders J., Altfeld M. (2004). Increased Natural Killer Cell Activity in Viremic HIV-1 Infection. J. Immunol..

[67] Penack O., Gentilini C., Fischer L., Asemissen A.M., Scheibenbogen C., Thiel E., Uharek L. (2005). CD56dimCD16neg Cells Are Responsible for Natural Cytotoxicity against Tumor Targets. Leukemia.

[68] Wijaya R.S., Read S.A., Truong N.R., Han S., Chen D., Shahidipour H., Fewings N.L., Schibeci S., Azardaryany M.K., Parnell G.P. (2021). HBV Vaccination and HBV Infection Induces HBV-Specific Natural Killer Cell Memory. Gut.

[69] Saraiva M., Vieira P., O’Garra A. (2020). Biology and Therapeutic Potential of Interleukin-10. J. Exp. Med..

[70] Schlums H., Cichocki F., Tesi B., Theorell J., Beziat V., Holmes T.D., Han H., Chiang S.C.C., Foley B., Mattsson K. (2015). Cytomegalovirus Infection Drives Adaptive Epigenetic Diversification of NK Cells with Altered Signaling and Effector Function. Immunity.

[71] Lyons A.B. (2000). Analysing Cell Division in Vivo and in Vitro Using Flow Cytometric Measurement of CFSE Dye Dilution. J. Immunol. Methods.

[72] Pantaleo G., Harari A. (2006). Functional Signatures in Antiviral T-Cell Immunity for Monitoring Virus-Associated Diseases. Nat. Rev. Immunol..

[73] White M.J., Nielsen C.M., Mcgregor R.H.C., Riley E.M., Goodier M.R. (2014). Differential Activation of CD57-Defined Natural Killer Cell Subsets during Recall Responses to Vaccine Antigens. Immunology.

[74] Liu B., Yang G.X., Sun Y., Tomiyama T., Zhang W., Leung P.S.C., He X.S., Dhaliwal S., Invernizzi P., Gershwin M.E. (2022). Decreased CD57 Expression of Natural Killer Cells Enhanced Cytotoxicity in Patients with Primary Sclerosing Cholangitis. Front. Immunol..

[75] Libri V., Schulte D., van Stijn A., Ragimbeau J., Rogge L., Pellegrini S. (2008). Jakmip1 Is Expressed upon T Cell Differentiation and Has an Inhibitory Function in Cytotoxic T Lymphocytes. J. Immunol..

[76] Liu L.L., Landskron J., Ask E.H., Enqvist M., Sohlberg E., Traherne J.A., Hammer Q., Goodridge J.P., Larsson S., Jayaraman J. (2016). Critical Role of CD2 Co-Stimulation in Adaptive Natural Killer Cell Responses Revealed in NKG2C-Deficient Humans. Cell Rep..

[77] Forrest C., Chase T.J.G., Cuff A.O., Maroulis D., Motallebzadeh R., Gander A., Davidson B., Griffiths P., Male V., Reeves M. (2023). Control of Human Cytomegalovirus Replication by Liver Resident Natural Killer Cells. Nat. Commun..

[78] Wang E.C.Y., Pjechova M., Nightingale K., Vlahava V.M., Patel M., Ruckova E., Forbes S.K., Nobre L., Antrobus R., Roberts D. (2018). Suppression of Costimulation by Human Cytomegalovirus Promotes Evasion of Cellular Immune Defenses. Proc. Natl. Acad. Sci. USA.

[79] Rölle A., Halenius A., Ewen E.M., Cerwenka A., Hengel H., Momburg F. (2016). CD2–CD58 Interactions Are Pivotal for the Activation and Function of Adaptive Natural Killer Cells in Human Cytomegalovirus Infection. Eur. J. Immunol..

[80] Ahopelto K., Böckelman C., Hagström J., Koskensalo S., Haglund C. (2016). Transketolase-like Protein 1 Expression Predicts Poor Prognosis in Colorectal Cancer. Cancer Biol. Ther..

[81] Pal M., Schwab L., Yermakova A., Mace E.M., Claus R., Krahl A.C., Woiterski J., Hartwig U.F., Orange J.S., Handgretinger R. (2017). Tumor-Priming Converts NK Cells to Memory-like NK Cells. Oncoimmunology.

[82] Shemesh A., Su Y., Calabrese D.R., Chen D., Arakawa-Hoyt J., Roybal K.T., Heath J.R., Greenland J.R., Lanier L.L. (2022). Diminished Cell Proliferation Promotes Natural Killer Cell Adaptive-like Phenotype by Limiting FcεRIγ Expression. J. Exp. Med..

[83] Tarragó D., González I., González-Escribano M.F. (2022). HLA-E Restricted Cytomegalovirus UL40 Peptide Polymorphism May Represent a Risk Factor Following Congenital Infection. BMC Genom..

[84] Martín-Villa J.M., Vaquero-Yuste C., Molina-Alejandre M., Juarez I., Suárez-Trujillo F., López-Nares A., Palacio-Gruber J., Barrera-Gutiérrez L., Fernández-Cruz E., Rodríguez-Sainz C. (2022). HLA-G: Too Much or Too Little? Role in Cancer and Autoimmune Disease. Front. Immunol..

[85] Le Discorde M., Moreau P., Sabatier P., Legeais J.M., Carosella E.D. (2003). Expression of HLA-G in Human Cornea, an Immune-Privileged Tissue. Hum. Immunol..

[86] LeBouder F., Khoufache K., Menier C., Mandouri Y., Keffous M., Lejal N., Krawice-Radanne I., Carosella E.D., Rouas-Freiss N., Riteau B. (2009). Immunosuppressive HLA-G Molecule Is Upregulated in Alveolar Epithelial Cells after Influenza A Virus Infection. Hum. Immunol..

[87] Amiot L., Vu N., Rauch M., L’Helgoualc’H A., Chalmel F., Gascan H., Turlin B., Guyader D., Samson M. (2014). Expression of HLA-G by Mast Cells Is Associated with Hepatitis C Virus-Induced Liver Fibrosis. J. Hepatol..

[88] Lafon M., Prehaud C., Megret F., Lafage M., Mouillot G., Roa M., Moreau P., Rouas-Freiss N., Carosella E.D. (2005). Modulation of HLA-G Expression in Human Neural Cells after Neurotropic Viral Infections. J. Virol..

[89] Zhao M., Zhang R., Xu X., Liu Y., Zhang H., Zhai X., Hu X. (2013). IL-10 Reduces Levels of Apoptosis in Toxoplasma Gondii-Infected Trophoblasts. PLoS ONE.

