# Supplementary material for: Autologous T-Cell-Free Antigen Presentation System Unveils hCMV-Specific NK Cell Response
Source: Cells. 2024 Mar 17;13(6):530. doi: 10.3390/cells13060530 (PMC10969127; doi:10.3390/cells13060530)
Supplement: Supplementary file 1 [file cells-13-00530-s001.zip › cells-2896551-supplementary.pdf]

Supplementary Material

Supplementary Figures

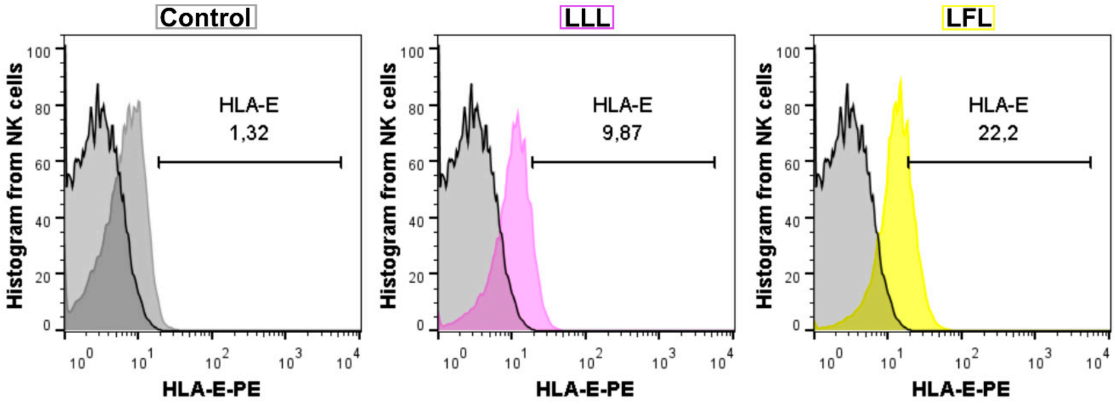

Supplementary Figure S1. Representative cytometric data of HLA-E surface expression without and after stimulation with LLL/LFL in NK cells.

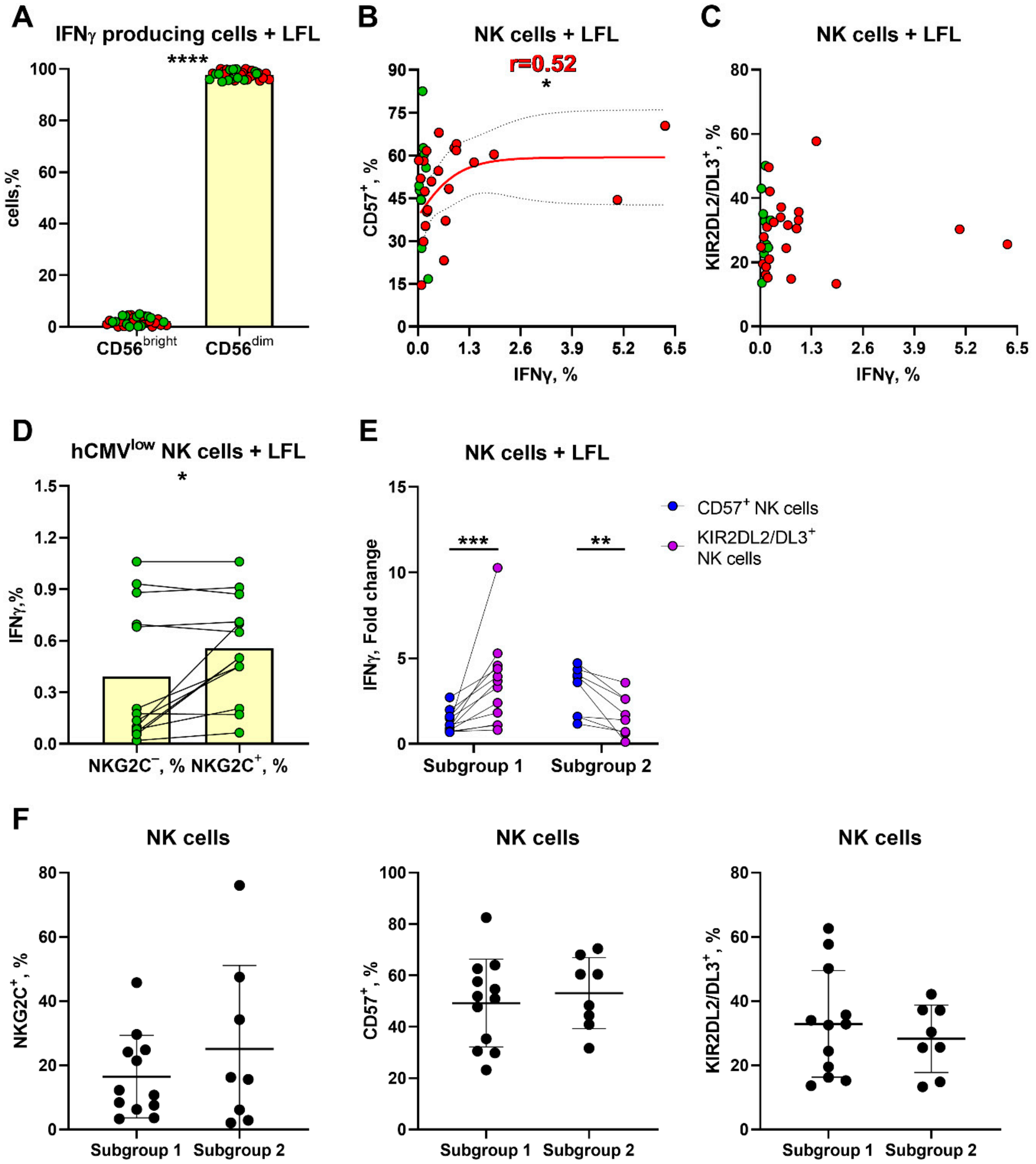

Supplementary Figure S2. IFNγ production by NK cells upon stimulation with the LFL peptide. **(A)** Comparison of the proportions of CD56^{bright} and CD56^{dim} NK cells producing IFNγ (*n*=34). **(B)** Spearman correlation analysis of CD57 expression and IFNγ production by NK cells stimulated with LFL in all studied groups, presented using a nonlinear regression model (*n*=34). **(C)** Spearman correlation analysis of KIR2DL2/DL3 expression and IFNγ production by NK cells stimulated with LFL in all studied groups (*n*=34). **(D)** Comparison of IFNγ production in NKG2C^{+} and NKG2C$^{–}$ NK cells in samples stimulated with LFL in the hCMV^{low} group (*n*=13). **(E)** Fold change of IFNγ production by CD57^{+} and KIR2DDL2DL3^{+} NK cells, separated into two subgroups (subgroup 1 –

n=12, subgroup 2 – *n*=8 of hCMV^{high} donors). **(F)** The percentage of NKG2C+, CD57+, KIR2DL2/DL3^{+} NK cells in two subgroups, identified in (C) (subgroup 1 – *n*=12, subgroup 2 – *n*=8 of hCMV^{high} donors). The color of the symbols corresponds to the groups, where green is hCMV^{low}, red is hCMV^{high}. NK cells were stimulated with the LFL peptide (VMAPRTLFL). Data are presented as symbols representing the mean of two replicates (B,C), mean values with lines connecting symbols for each donor(D,E), mean±SD (A,F). The paired Wilcoxon test was used to analyze the data (D,E), and the T test was used for unpaired data (A,F). *p<0.05, **p<0.01, ***p<0.001.

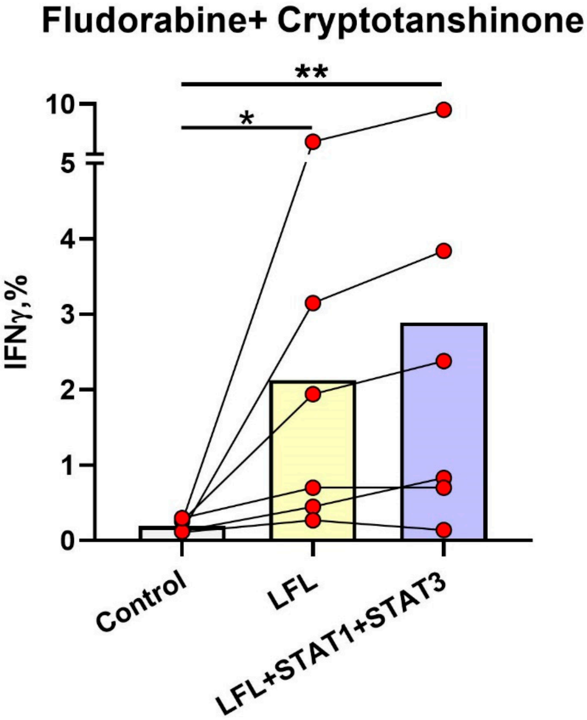

Supplementary Figure S3. Effect of simultaneous addition of STAT1 (Fludorabine) and STAT3 (Cryptotanshinone) inhibitors on the level of IFNγ production in NK cells of hCMV^{high} donors (*n*=6). Data are presented as mean values with lines connecting symbols for each donor. Each symbol represents the mean of two replicates. The paired Friedman statistical test was used to analyze the data. *p<0.05, **p<0.01.

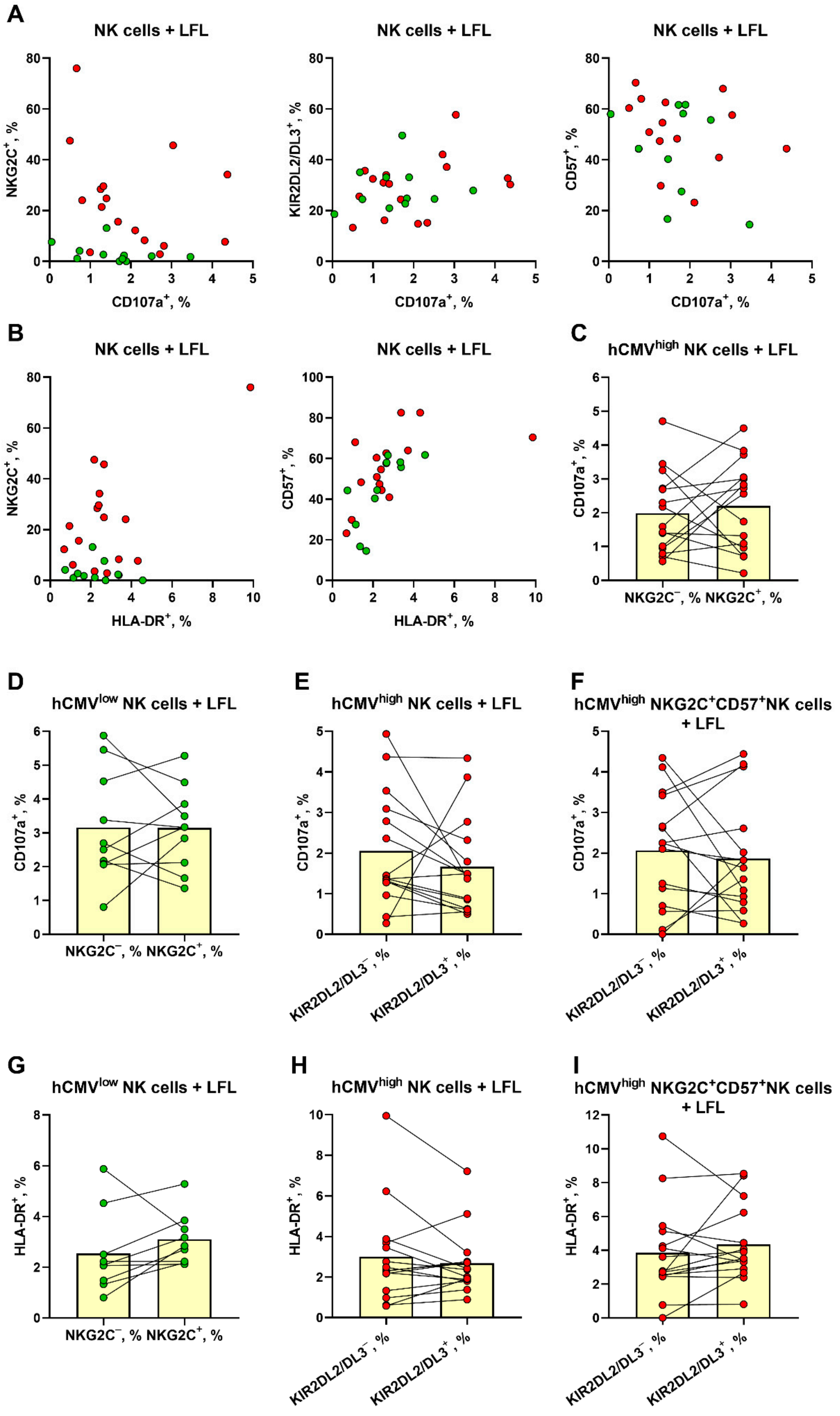
A
NK cells + LFL
NKG2C+, %
CD107a+, %
NK cells + LFL
KIR2DL2/DL3+, %
CD107a+, %
NK cells + LFL
CD57+, %
CD107a+, %
B
NK cells + LFL
NKG2C+, %
HLA-DR+, %
NK cells + LFL
CD57+, %
HLA-DR+, %
C
hCMVhigh NK cells + LFL
CD107a+, %
NKG2C−, % NKG2C+, %
D
hCMVlow NK cells + LFL
CD107a+, %
NKG2C−, % NKG2C+, %
E
hCMVhigh NK cells + LFL
CD107a+, %
KIR2DL2/DL3−, %
KIR2DL2/DL3+, %
F
hCMVhigh NKG2C+CD57+NK cells + LFL
CD107a+, %
KIR2DL2/DL3−, %
KIR2DL2/DL3+, %
G
hCMVlow NK cells + LFL
HLA-DR+, %
NKG2C−, % NKG2C+, %
H
hCMVhigh NK cells + LFL
HLA-DR+, %
KIR2DL2/DL3−, %
KIR2DL2/DL3+, %
I
hCMVhigh NKG2C+CD57+NK cells + LFL
HLA-DR+, %
KIR2DL2/DL3−, %
KIR2DL2/DL3+, %

Supplementary Figure S4. Degranulation and activation of NK cells upon stimulation with the LFL peptide. **(A)** Spearman correlation analysis of NKG2C, CD57, KIR2DL2/DL3 expression and level of CD107a expression by NK cells stimulated with LFL in all studied groups (*n*=27). **(B)** Spearman correlation analysis of NKG2C, CD57 expression and level of HLA-DR expression by NK cells stimulated with LFL in all studied groups (*n*=27). **(C)** Comparison of CD107a expression by $NKG2C^+$ and $NKG2C^-$ NK cells in samples stimulated with LFL in $hCMV^{high}$ group (n=16). **(D)** Comparison of CD107a expression by $NKG2C^+$ and $NKG2C^-$ NK cells in samples stimulated with LFL in $hCMV^{low}$ group (*n*=11). **(E)** Comparison of CD107a expression by KIR2DL2/$DL3^+$ and KIR2DL2/$DL3^-$ NK cells in samples stimulated with LFL in $hCMV^{high}$ group (*n*=16). **(F)** Comparison of CD107a expression by KIR2DL2/$DL3^+$ and KIR2DL2/$DL3^-$ NKG2C+CD57+NK cells in samples stimulated with LFL in $hCMV^{high}$ group (*n*=16). **(G)** Comparison of HLA-DR expression by $NKG2C^+$ and $NKG2C^-$ NK cells in samples stimulated with LFL in $hCMV^{low}$ group (*n*=11). **(H)** Comparison of HLA-DR expression by KIR2DL2/$DL3^+$ and KIR2DL2/$DL3^-$ NK cells in samples with stimulated LFL in $hCMV^{high}$ group (*n*=16). **(I)** Comparison of HLA-DR expression by KIR2DL2/$DL3^+$ and KIR2DL2/$DL3^-$ $NKG2C^+CD57^+$NK cells in samples stimulated with LFL in $hCMV^{high}$ group (*n*=16). The color of the symbols corresponds to the groups, wherein green – $hCMV^{low}$, red – $hCMV^{high}$. The color of the boxes corresponds to stimulation conditions, where gray is no stimulation and light yellow – stimulation with the LFL peptide (VMAPRTLFL). Data are presented as mean values with lines connecting symbols for each donor. Each symbol represents the mean of two replicates.

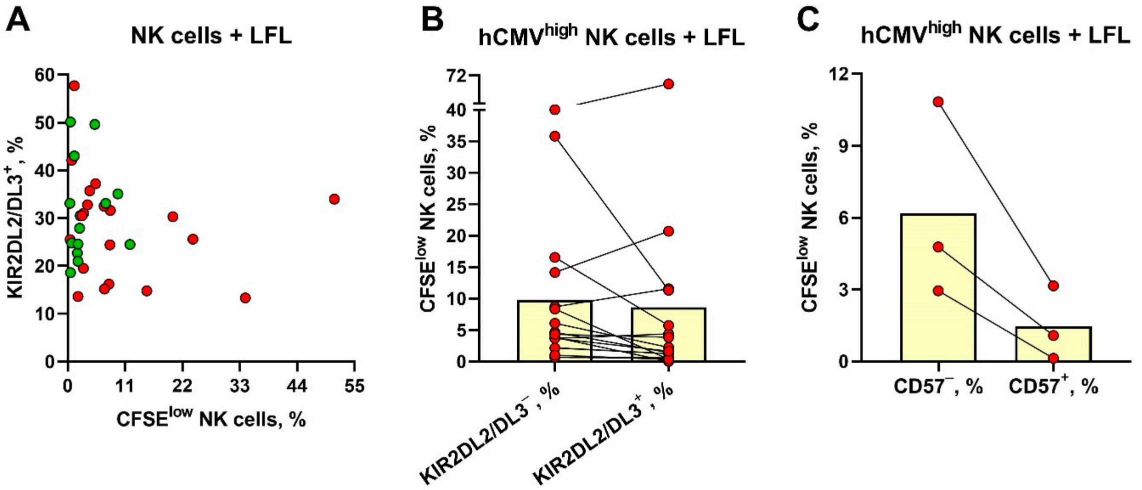

Supplementary Figure S5. Proliferative potential of NK cells after 7 days of incubation with the LFL peptide, and following characterization of $CFSE^{low}$ NK cells. **(A)** Spearman correlation analysis of KIR2DL2/DL3 expression and the percentage of $CFSE^{low}$ NK cells stimulated with LFL in all studied groups (*n*=34). **(B)** Comparison of the percentage of $CFSE^{low}$ NK cells in KIR2DL2/$DL3^+$ and KIR2DL2/$DL3^-$ NK cells in samples stimulated with LFL in $hCMV^{high}$ group (*n*=16). **(C)** Comparison of the percentage of $CFSE^{low}$ NK cells in $CD57^+$ and $CD57^-$ NK cells in samples stimulated with LFL in $hCMV^{high}$ group (*n*=3). The color of the symbols corresponds to the groups, where green is $hCMV^{low}$, red is $hCMV^{high}$. The color of the boxes corresponds to stimulation conditions, wherein gray – no stimulation and light yellow – stimulation with the LFL peptide (VMAPRTLFL). Data are presented

as mean values with lines connecting symbols for each donor. Each symbol represents the mean of two replicates.

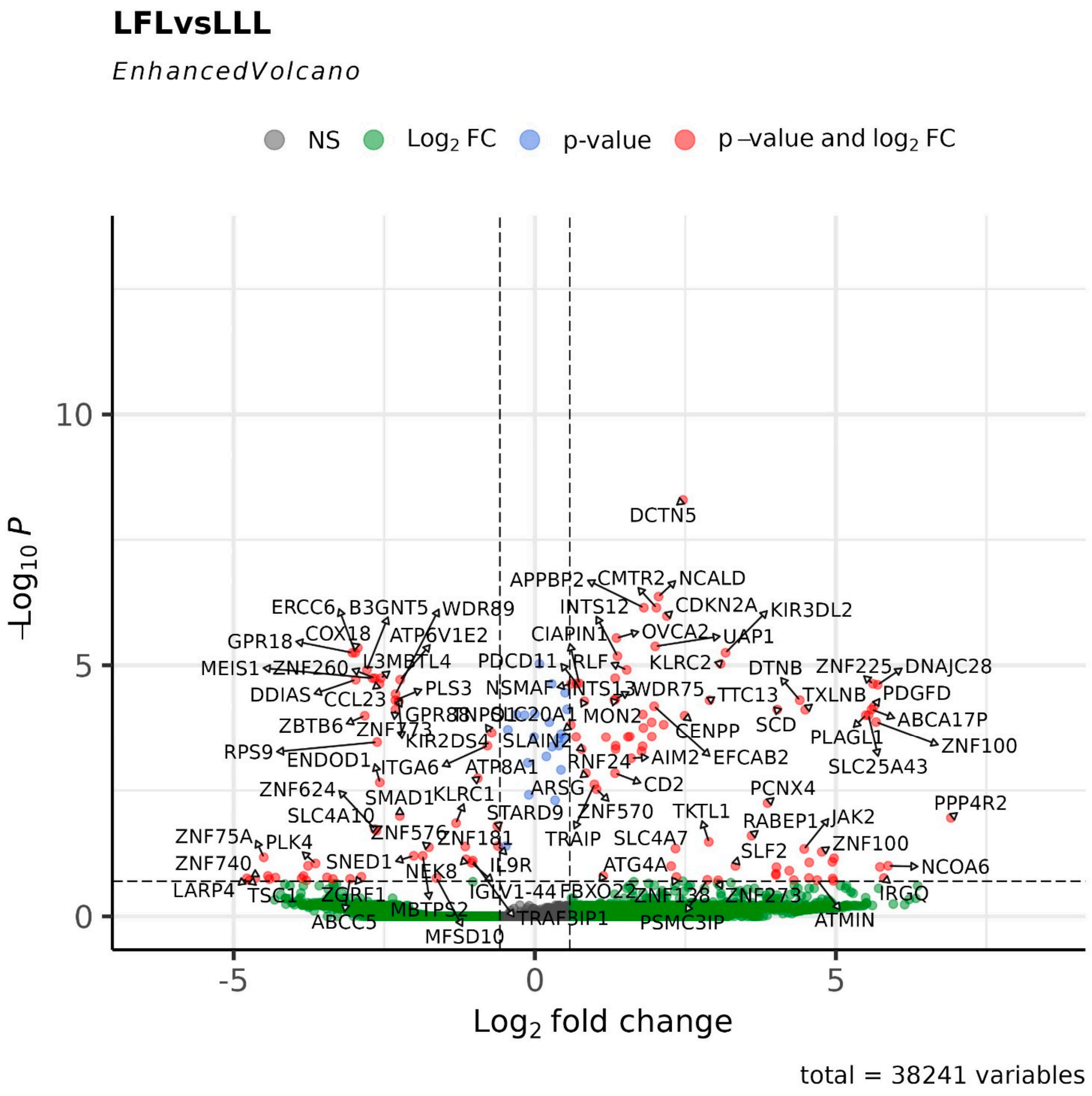

Supplementary Figure S6. Volcano plot comparing LFL-stimulated with LLL-stimulated CFSE^{low} NK cells significant 96 out of 130 up-regulated and down-regulated genes are labeled.
